# Unveiling Cortical Criticality Changes along the Prodromal to the Overt Continuum of Alpha-Synucleinopathy

**DOI:** 10.1523/JNEUROSCI.1871-24.2025

**Published:** 2025-07-03

**Authors:** Monica Roascio, Sheng H. Wang, Vladislav Myrov, Felix Siebenhühner, Rosella Trò, Pietro Mattioli, Francesco Famà, Silvia D. Morbelli, Matteo Pardini, Beatrice Orso, J. Matias Palva, Dario Arnaldi, Gabriele Arnulfo

**Affiliations:** ^1^Department of Informatics, Bioengineering, Robotics and System Engineering (DIBRIS), University of Genoa, Genoa 16145, Italy; ^2^Neuroscience Center, Helsinki Institute of Life Science (HiLife), University of Helsinki, Helsinki FI-00014, Finland; ^3^Department of Neuroscience and Biomedical Engineering, Aalto University, Espoo FI-00076, Finland; ^4^CEA, NeuroSpin, Gif-Sur-Yvette 91190, France; ^5^MIND, Inria, Palaiseau 91120, France; ^6^BioMag Laboratory, HUS Medical Imaging Center, Helsinki 00290, Finland; ^7^Department of Neurosciences, Rehabilitation, Ophthalmology, Genetics, Maternal and Children's Sciences (DINOGMI), University of Genoa, Genoa 16132, Italy; ^8^Neurophysiology Unit, IRCCS Ospedale Policlinico San Martino, Genoa 16132, Italy; ^9^Nuclear Medicine Unit, Città della Salute e della Scienza di Torino, Turin 10126, Italy; ^10^Department of Medical Sciences, University of Turin, Turin 10124, Italy; ^11^Clinical Neurology, IRCCS Ospedale Policlinico San Martino, Genoa 16132, Italy; ^12^RAISE Ecosystem, Genoa 16122, Italy

**Keywords:** cortical excitation–inhibition balance, critical cortical dynamics, idiopathic REM sleep behavior disorder, pathophysiology

## Abstract

Patients with idiopathic/isolated REM sleep behavior disorder (iRBD) are in the prodromal stage of alpha-synucleinopathies. Neurodegeneration early affects subcortical structures, including the substantia nigra, in iRBD patients. However, it remains unclear whether there is also an early neurodegeneration process affecting the cerebral cortex. We investigated whether EEG-derived metrics for aberrant cortical dynamics and imbalanced excitation–inhibition (E/I) correlate with disease severity in iRBD patients, aiming to better understand the pathophysiology progression from the prodromal to the overt stage of alpha-synucleinopathies. We retrospectively analyzed resting-state EEG recordings, as a marker of cortical function, and presynaptic dopaminergic imaging, a marker of subcortical function from 59 iRBD patients (9 female) who underwent longitudinal clinical evaluation alongside 46 age-matched healthy controls (22 female). We assessed power-law scaling in long-range temporal correlations (LRTCs), neuronal bistability, and functional E/I balance from the resting-state sensor EEG data and then correlated these to large-scale synchrony, nigrostriatal dopaminergic function, and clinical data. Compared with the control group, patients showed higher LRTCs and bistability in 2–7 Hz oscillations. Patients who developed parkinsonism/dementia exhibited hyperexcitability in 5–7 Hz compared with those who did not. This was also correlated with stronger phase synchrony. Both hyperexcitability in 5–7 Hz and bistability in 2–4 Hz negatively associated with nigrostriatal dopaminergic impairment. The iRBD patients, especially those closer to phenoconversion to parkinsonism or dementia, show clear aberrant cortical dynamics and hyperexcitability alongside substantia nigra impairment, suggesting that neurodegeneration in the prodromal stages affects both subcortical structures and cortical dynamics.

## Significance Statement

During prodromal stage of alpha-synucleinopathies, symptoms are heterogeneous and occur with unpredictable onset, but most importantly, the neurodegenerative pathological mechanisms are not well understood, in particular the cortical involvement in the neurodegeneration process. Leveraging an unprecedented longitudinal dataset combining dopamine-depletion assessment, clinical evaluations, and resting-state high-density EEG recordings, we found that mild bistability and hyperexcitability—hallmarks of a system on the verge of catastrophic transition—characterized iRBD patients near phenoconversion up to ∼3 years before conversion. These findings suggest that cortical dysfunction is an early event along the prodromal to overt alpha-synucleinopathy neurodegeneration continuum. This significantly advances the understanding of the pathophysiological neurodegeneration process in the prodromal stage of alpha-synucleinopathies and in the transition from the prodromal to overt stage.

## Introduction

Abnormal accumulation of alpha-synuclein protein aggregates in neurons or glial cells characterizes alpha-synucleinopathies, leading to conditions such as Parkinson's disease (PD), dementia with Lewy bodies (DLB), and multiple system atrophy (MSA; Spillantini et al., 1997). Clinical diagnoses are typically made when overt syndromes like PD and DLB manifest, as these diseases present with clear disabling symptoms. The neurodegeneration process begins several years before the emergence of parkinsonism/dementia (Arnaldi et al., 2021). REM sleep behavior disorder (RBD) is considered an early biomarker of alpha-synucleinopathy (Heinzel et al., 2019; McKeith et al., 2020), and most iRBD patients would develop parkinsonism/dementia within 12 years from the initial diagnosis (Galbiati et al., 2019; Postuma et al., 2019; Arnaldi et al., 2021; Mattioli et al., 2023). This makes idiopathic/isolated RBD (iRBD) patients (i.e., patients without overt parkinsonism or dementia) an ideal population to study brain changes that occur before the onset of full-blown alpha-synucleinopathies (Postuma et al., 2019).

In this study, we employed novel biomarkers inspired by brain criticality and complex-systems theory to study the EEG recording from iRBD patients. The “brain criticality hypothesis” posits that the brain gains functional benefits from operating near a phase transition between hypersynchrony (supercritical) and asynchrony (subcritical) phases (Beggs, 2008; Chialvo, 2010). Evidence points to the healthy brain operating in slightly subcritical or inhibited condition (Priesemann et al., 2013; Wilting and Priesemann, 2019; Fuscà et al., 2023), but still reaping the benefits of critical dynamics. In this regime, the oscillatory activity of the neuronal field potentials is characterized by stronger long-range temporal correlations (LRTCs), optimal E/I balance (Kinouchi and Copelli, 2006), and intermediate levels of neuronal synchronization (Fuscà et al., 2023). A continuous reduction of LRTCs and increasing excitability characterize the progression of Alzheimer's disease (AD) patients from subclinical (subjective cognitive decline) to prodromal (mild cognitive impairment—MCI) to overt (dementia) stages (Javed et al., 2022; van Nifterick et al., 2023).

Recent modeling (Freyer et al., 2012; di Santo et al., 2018; Wang et al., 2023) and empirical evidence (Freyer et al., 2009; Wang et al., 2024a) suggest that near the critical phase transition neurons can also show bistable dynamics. Mild neuronal bistability reflects beneficial excitability and positively correlates with cognitive performance in healthy subjects (Wang et al., 2023), whereas strong bistability thought to be associated with pathological conditions and has been proposed as a biomarker for the epileptogenic zone (Wang et al., 2024a,b; Burlando et al., 2025). Alpha-synucleinopathies, especially PD, are believed to early affect subcortical structures and later the cerebral cortex (Braak et al., 2003). Other models suggest an early involvement of the cerebral cortex, especially when considering all Lewy body disorders phenotypes, including DLB and PD with cognitive impairment and dementia (Borghammer, 2023). Although subcortical abnormalities have been extensively studied in iRBD patients, cerebral cortical dysfunction has been poorly investigated (Miglis et al., 2021). Compared with healthy subjects, iRBD patients exhibit a slowing of the alpha peak (Fantini et al., 2003; Iranzo et al., 2010) and weaker neuronal synchronization in the delta band at diagnosis (Sunwoo et al., 2017). Following disease progression, neuronal synchronization further increases in the alpha band (Roascio et al., 2022). These previous results suggest that a cortical dysfunction may be already present.

**Figure 1.**
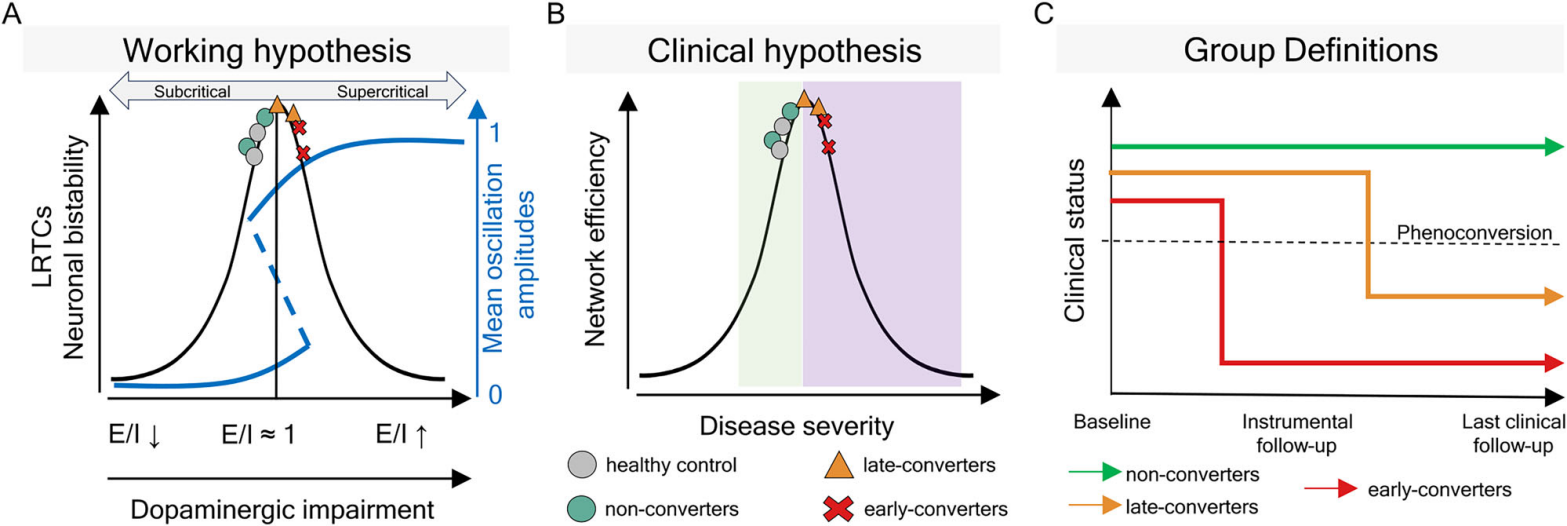
Study hypothesis. ***A***, Working hypothesis: the relationship between long-range temporal correlations (LRTCs, i.e., power-law scaling in oscillations), E/I balance, and neuronal bistability that can be assessed with resting-state hdEEG from the subjects (markers). ***B***, Clinical hypothesis. The green shade is the physiological range; the purple shade is the pathological range. Markers represent patients and control subjects. ***C***, Grouping of iRBD patients by increasing disease severity. Non-converters remained clinically stable throughout the study. Late-converters developed parkinsonism or dementia after the first instrumental follow-up. Early-converters developed parkinsonism/dementia before the first instrumental follow-up.

We hypothesized that iRBD patients show altered critical cortical dynamics and hyperexcitability, similar to how MCI affects cortical dynamics before AD (Fig. 1*A*,*B*). We expected the extent of these alterations to be highly correlated with disease severity. We investigated how LRTCs, E/I balance, and cortical bistability differed in iRBD patients compared with healthy subjects assessed using high-density EEG data. We investigated how critical dynamics correlated with biological (presynaptic dopaminergic imaging) and clinical (motor and cognitive assessment) severity in iRBD patients who developed parkinsonism/dementia over time compared with those who remained clinically stable (Fig. 1*C*). Finally, we investigated whether E/I (im)balance is correlated with phase synchronization in iRBD patients.

## Materials and Methods

### Sample characteristics

In this study, we enrolled 62 iRBD patients (9 female; mean age 69.58 ± 7.21 years) who underwent baseline high-density (64 channels) EEG (hdEEG) resting-state recordings, dopamine transporter (DaT) imaging with [^123^I]FP-CIT-SPECT, and a comprehensive clinical assessment including (1) the Mini-Mental State Examination (MMSE) as a global measure of cognitive impairment; (2) the Movement Disorder Society-sponsored revision of the Unified Parkinson's Disease Rating Scale, motor section (MDS-UPDRS-III) to evaluate the presence of parkinsonian signs; and (3) clinical interviews and questionnaires for activities of daily living (ADL) and instrumental ADL to exclude dementia. The diagnosis of iRBD was confirmed by overnight video-polysomnography, according to current criteria (American Academy of Sleep Medicine, 2014). Idiopathic RBD patients underwent general and neurological examinations to exclude other neurological and psychiatric disorders. The duration of RBD prior to diagnosis was 41.6 ± 34.2 months.

All patients underwent clinical follow-ups every 6 months, including motor and cognitive assessments and 50% of patients (31 men, mean age 72.41 ± 7.05) underwent instrumental follow-up, evaluating hdEEG (*n* = 31) and DaT-SPECT (*n* = 28) after ∼2 years (25.79 ± 12.45 months) from the diagnosis of iRBD.

Within the ∼2 years time window of this study (23.82 ± 18.13 months from iRBD diagnosis), 18 (29%) iRBD patients (4 female, mean age 73.35 ± 6.05) phenoconverted (8 to PD and 10 to DLB). Of note, 8 out of 18 iRBD patients had already developed overt parkinsonism/dementia at the time of the instrumental follow-up (early-converter iRBD patients), and the remaining 10 had developed overt parkinsonism/dementia at the last clinical follow-up (late-converter iRBD patients). The phenoconversion to Parkinson's disease, dementia with Lewy bodies, or multiple system atrophy was defined based on current clinical criteria (Gilman et al., 2008; Postuma et al., 2015; McKeith et al., 2017).

We grouped patients as follows: (1) non-converter iRBD if patients had not developed parkinsonism/dementia at the last available clinical follow-up; (2) late-converter iRBD if patients were still free from parkinsonism/dementia at the first time point, that is, the instrumental follow-up, but then phenoconverted between the first time point and the last one (i.e., last clinical follow-up); and (3) early-converter iRBD if the phenoconversion diagnosis was made before the instrumental follow-up (Fig. 1*C*, Table 1).

**Table 1.** Demographic and clinical measurements

Baseline	HC	ncRBD	lRBD	eRBD	*p*
Subjects (#)	46	41	10	8	n.a.
PD (#)	n.a.	n.a.	3	5	n.a
DLB (#)	n.a.	n.a.	7	3	n.a.
Age	70.50 ± 10.21	68.10 ± 7.23	71.60 ± 6.31	70.50 ± 6.16	*p* = 0.276
Sex (F/M)	22/24	5/36	2/8	2/6	*p* = 0.003
Education	n.a.	9.59 ± 3.43	11.00 ± 4.58	9.88 ± 4.40	*p* = 0.849
MMSE	28.48 ± 2.29	28.49 ± 1.31	26.80 ± 3.77	26.75 ± 2.71	*p*_HC,lRBD_ = 0.043 *p*_HC,eRBD_ = 0.045 *p*_HC,ncRBD_ = 0.996
MDS-UPDRS-III	0	1.53 ± 3.36	1.40 ± 2.50	3.63 ± 2.33	*p*_HC,lRBD_ = 0.998 *p*_HC,eRBD_ = 0.998 *p*_HC,ncRBD_ = 0.998
Follow-up	HC	ncRBD	lRBD	eRBD	*p*
Subjects (#)	n.a.	17	6	8	n.a.
PD (#)	n.a.	n.a.	1	5	n.a.
DLB (#)	n.a.	n.a.	5	3	n.a.
Age	n.a.	72.24 ± 7.53	70.50 ± 3.50	72.00 ± 5.83	*p* = 0.707
Sex (F/M)	n.a.	3/14	1/5	2/6	*p* = 0.898
Education	n.a.	10.20 ± 3.89	10.25 ± 4.44	15.00 ± 2.00	*p* = 0.334
MMSE	n.a.	28.41 ± 1.70	28.00 ± 1.41	24.88 ± 4.64	*p*_ncRBD,lRBD_ = 0.219 *p*_ncRBD,eRBD_ < 0.0001
MDS-UPDRS-III	n.a.	1.29 ± 1.79	7.17 ± 8.57	10.88 ± 15.81	*p*_ncRBD,lRBD_ < 0.0001 *p*_ncRBD,eRBD_ < 0.0001

Demographic and clinical data for non-converter, late-converter, and early-converter iRBD patients at baseline and at instrumental follow-up for iRBD patients alone. eRBD, early-converter iRBD patients; DLB, dementia with Lewy bodies; HC, healthy controls; MMSE, mini-mental examination test; MDS-UPDRS-III, movement disorder society-sponsored revision of the Unified Parkinson's disease rating scale, motor section; lRBD, late-converter iRBD patients; ncRBD, non-converter iRBD patients; PD, Parkinson's disease.

As a control dataset, we included 48 age-matched healthy control (HC) subjects (23 female, 70.25 ± 10.26) who underwent baseline clinical evaluation (i.e., MMSE) and resting-state hdEEG recording as part of a previous voluntary program in our institution.

The study was conducted according to the declaration of Helsinki, and all participants gave informed consent before entering the study, which the local ethics committee approved.

### hdEEG collection and preprocessing

The first hdEEG recording was carried out within 3 months of diagnosis. All subjects underwent hdEEG recording during relaxed wakefulness late in the morning to minimize drowsiness. For each session, the acquisition protocol consisted of ∼22.71 ± 3.71 min of resting-state subdivided into eyes-open, eyes-closed, and hyperventilation condition. We used the Galileo system (EBNeuro) to acquire bandpass (0.3–100 Hz) signals from 64 electrodes (10–10 International System) at a sampling rate of 512 Hz, where the reference electrode and ground were Fpz and Oz, respectively. Electrode impedances were monitored and kept below 5 kOhm.

We filtered the time series with a notch filter (order 2) to remove power line noise (50 Hz). We rejected the channels with a high percentage of artifacts (number of rejected channels 2.57 ± 2.43, mean ± standard deviation). We used independent component analysis and visual inspection to reduce the number of physiological and instrumentation artifacts, such as blinks, lateral eye movements, muscle artifacts, drowsiness, and electrode pop. We filtered the time series with a Finite Impulse Response bandpass filter (1–80 Hz, Kaiser window, order 1858). We interpolated bad channels using spline interpolation (kernel size: 4 cm). For all clean sensors, we applied the scalp current density (SCD) transformation based on spherical spline surface Laplacian (lambda 0.00001, order 4; Perrin et al., 1989). For the following analysis, we selected ∼9.68 ± 3.71 min of eye-closed resting-state condition.

After hdEEG preprocessing, we excluded three iRBD patients at baseline and two HC subjects due to excessive artifactual activity in the EEG recording, which left <1 min of eye-closed resting-state data after cleaning. For the following analysis, we retained 59 iRBD patients and 46 HC subjects at baseline (Table 1).

### hdEEG metrics

**Figure 2.**
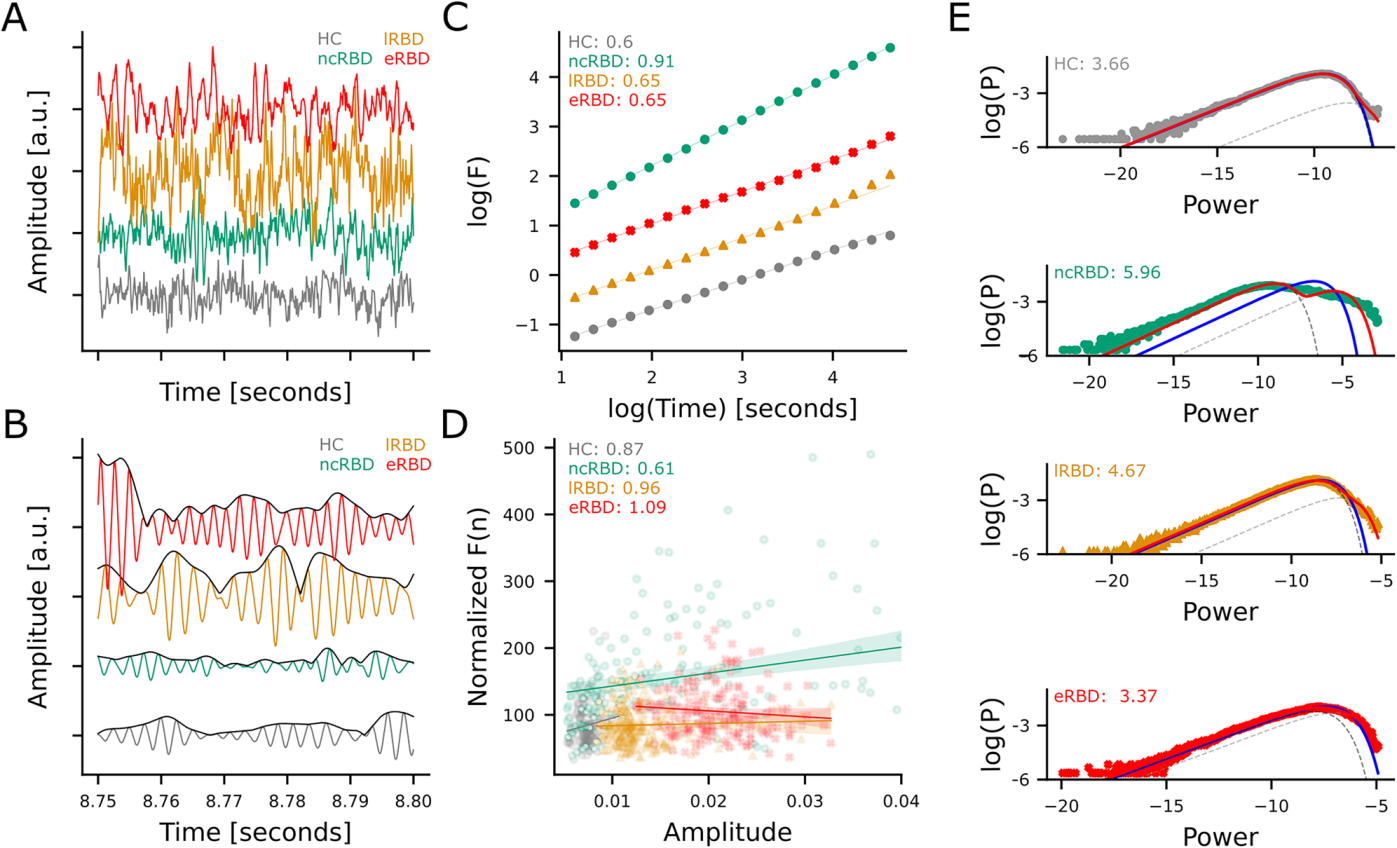
Operationalizations of the criticality assessments from resting-stage EEG. ***A***, EEG single-channel broadband time series and their (***B***) Single-channel narrow-band (7 Hz) oscillations and their amplitude envelopes (black line). ***A***, ***B***, Trace color code: healthy subject (gray), a non-converter iRBD patient (green), a late-converter iRBD patient (orange), and an early-converter iRBD patient (red). ***C–E***, Illustration of criticality assessment for the traces shown in (***B***) with the same color code. ***C***, LRTCs were assessed using the detrended fluctuation scaling exponent, (***D***) functional E/I balance was assessed using the functional E/I index (fEI), and (***E***) neuronal bistability was assessed using the bistability index (BiS).

We analyzed the broadband SCD time series with a time–frequency decomposition using 30 log-spaced narrowband Morlet wavelets (*m* = 5) in the 2–70 Hz range (Fig. 2*A*,*B*; Tallon-Baudry et al., 1998; Torrence and Compo, 1998). For each time–frequency series, we estimated the detrended fluctuation analysis scaling exponent (DFA), the functional E/I ratio (fEI), the bistability index (BiS), and the weighted phase lag index (wPLI) as described below.

#### Detrended fluctuation analysis

Detrended fluctuation analysis (DFA) was used to investigate LRTCs. We quantified the scale-free decay of temporal correlations in the amplitude modulation of neuronal oscillations (Linkenkaer-Hansen et al., 2001) with the DFA scaling exponent, which is the slope of the fluctuation function in log-log plot (Fig. 2*C*). A DFA exponent in the range [0.5, 1] indicates positive temporal correlations while an exponent of 0.5 is characteristic of uncorrelated signal and an exponent below that indicates negative correlations (Hardstone et al., 2012).

#### Functional excitation–inhibition ratio

To quantify the E/I balance (Fig. 2*D*), we computed the functional excitation–inhibition ratio (fEI) ratio for windows with a fixed length of 40 cycles for each narrow-band signal and 80% overlap (Bruining et al., 2020). For each channel, we computed fEI only when the DFA scaling exponent was >0.6 because channels without LRTC do not exhibit covariation of amplitude and fluctuation function.

Systems operating at the critical transition should have fEI = 1, while those working in the inhibition- or excitation-dominated states should have an fEI < 1 or fEI > 1, respectively.

#### Bistability index

Cortical bistability represents a discontinuous transition between asynchronous and totally synchronous activity (Freyer et al., 2011), and high bistability is considered a sign of brain pathology. To evaluate cortical bistability (Fig. 2*E*), we quantified the bistability index (BiS) of a power time series *R*^2^ fitting its probability density function (PDF) with a single- and a bi-exponential model. The single-exponent model is defined as follows:

$$P_{R^{2}}(R^{2})\;=\;\gamma e^{-\gamma R^{2}},\;(1)$$

where γ is the exponent. The bi-exponential model is defined as follows:

$$P_{R^{2}}(R^{2})\;=\;\frac{1}{\left(\delta_{1}+\;\delta_{2}\right)}(\delta_{1}\gamma_{1}e^{-\gamma_{1}R^{2}}+\;\delta_{2}\gamma_{2}e^{-\gamma_{2}R^{2}}),\;(2)$$

where $\gamma_{1},\;\gamma_{2}$ are the two exponents and $\delta_{1}$, $\delta_{2}$ is a weighting factor. To assess the fitting of the two models, we used the Bayesian information criterion (BIC):

$$\mathrm{BIC}\;=\;ln(n)k\;-\;2ln(\hat{L}),$$

where *n* is the number of samples; $\hat{L}$ is the likelihood function; k is the number of free parameters in the model, i.e., for Equation 1, *k* = 1 and for Equation 2 and *k* = 4. A better-fitted model yields a small BIC value.

Next, we computed the difference in BIC between single- and bi-exponential fitting:

$$\mathrm{dBIC}=\mathrm{BI}C_{\exp}-\mathrm{BI}C_{\mathrm{biexp}},$$

Finally, the BiS is computed as:

$$\mathrm{BiS}\;=\;\mathrm{lo}g_{10}(\mathrm{dBIC})\;\;\mathrm{if}\;\mathrm{dBIC}\;>\;0\;,$$

BiS=0otherwise.

A BiS close to zero means that the single-exponential model is a more likely model for the observed time series, whereas, for a BiS >3, the most likely model for the observed time series is the bi-exponential model.

#### Weighted phase lag index

To quantify phase synchronization, we computed the weighted phase lag index (wPLI) for each pair of channels (Vinck et al., 2011). The wPLI is neither inflated by volume conduction as the phase-locking value (PLV) nor underestimating the true phase coupling as the imaginary part of the complex-valued PLV (Palva et al., 2018) and thus is ideal for EEG sensor level synchrony analysis intended here. The wPLI is limited to the range [0,1], where 0 represents the absence of synchronization. From the wPLI matrix, we estimated the eigenvector centrality, local clustering coefficient, and strength for each node (channel). The eigenvector centrality measures the influence of a node in a graph. The strength is the sum of the weights of links connected to the node. The local clustering coefficient measures the degree to which the nodes in a graph tend to cluster for any given node and is quantified by the fraction of how many possible triplets (i.e., three nodes connected together) are realized between the neighbors of a node.

For the statistical analysis, we grouped the frequency spectrum (2–70 Hz) in five frequency bands based on the canonical brain rhythms: delta (2–4 Hz), theta (5–7 Hz), alpha (8–13 Hz), beta (15–30 Hz), and gamma (30–70 Hz).

### Molecular imaging evaluation

Within 3 months after diagnosis, subjects with iRBD underwent [^123^I]FP-CIT-SPECT to measure the presynaptic dopaminergic function according to European Association of Nuclear Medicine guidelines (Darcourt et al., 2010; Morbelli et al., 2020). Reconstructed images were exported in the Analyze file format and processed by Basal Ganglia V2 software (Nobili et al., 2013) to compute specific to nondisplaceable binding ratios (SBRs), as detailed in a previous paper (Roascio et al., 2022). All patients were free from treatments that could have influenced DaT-SPECT findings.

### Statistical analysis

We performed a Kruskal–Wallis *H* test (*ɑ* = 0.05) to observe if demographic information (i.e., age, sex, and education) change across subject groups. We fitted a generalized linear model (GLM) to observe the difference in clinical scores between subject groups by adjusting for age and sex.

To quantify statistical difference in EEG measures between iRBD patients at baseline and HC subjects, we fitted a GLM on each EEG measures grouped in canonical frequency bands (i.e., delta: 2–4 Hz, theta: 5–7 Hz, alpha: 8–13 Hz, beta: 15–30 Hz, and low gamma: 30–70 Hz). We corrected for the multiple comparison using Bonferroni’s method (αcorrected < *α*/*N*, where *N* is the number of tests). To evaluate the magnitude of the difference between each two groups, we quantified the effect size using Cohen's *d*, which classified the effect size as small (*d* = 0.2), medium (*d* = 0.5), and large (*d* ≥ 0.8; Cohen, 1988). As above, we fitted a GLM on each EEG measures to investigate the statistical difference between baseline and follow-up recordings.

We fitted a linear mixed model (LMM) to investigate the relationship between hdEEG measures (i.e., DFA, fEI, and BiS)—averaged across channels and grouped in canonical frequency bands—and SBR values, adding age and sex as fixed effect. In this analysis, we included both baseline and follow-up visits; to consider the dependence between subjects with two visits, we added subjects as random effect. To evaluate the correlation between EEG features and clinical scores (i.e., MMSE and MDS-UPDRS-III), we used an LLM, adjusting for age and sex and considering subjects as a random effect as above. Finally, we explored the correlation between E/I balance and phase synchronization—both averaged across channels—using LMM, adding age and sex as fixed effect and subjects as random effect. We corrected for the multiple comparison using Bonferroni’s method (*α*_corrected_ < *α*/*N*, where *N* is the number of tests).

## Results

### Cortical neurodegeneration alters E/I balance in iRBD patients at baseline

We hypothesized that patients with iRBD already exhibited cortical neurodegeneration at the time of diagnosis, which would inhibit cortical dynamics compared with healthy individuals. To investigate this hypothesis, we quantified the spectral profiles (decomposition with 30 Morlet wavelets) of (1) the LRTCs with DFA, (2) the functional E/I ratio with fEI, and (3) the neuronal bistability with BiS.

**Figure 3.**
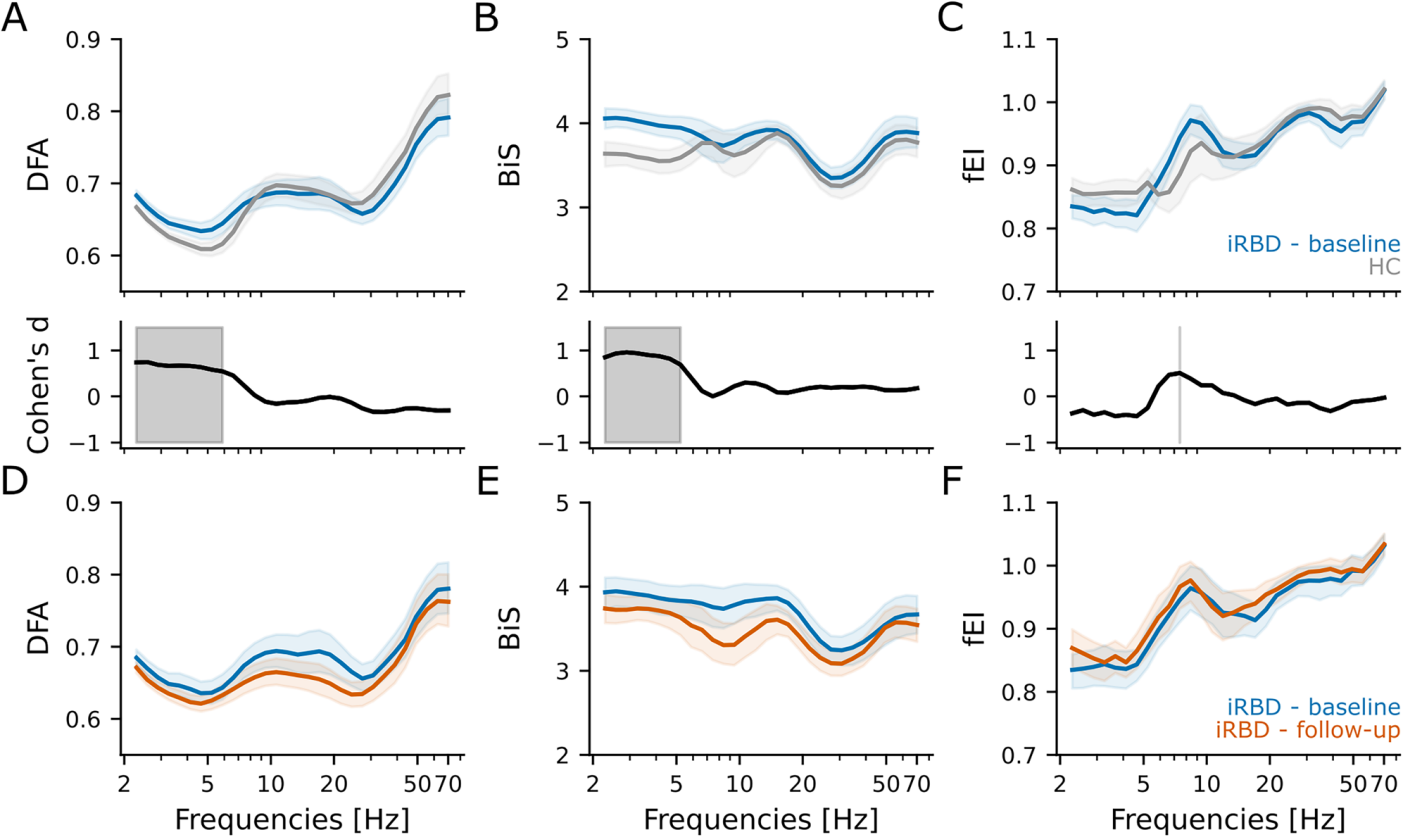
Stronger LRTCs and bistability in resting-state EEG characterize iRBD patients at baseline. Group-level averaged (top) and effect size of the difference estimated by Cohen's *d* (bottom) of (***A***) DFA exponent, (***B***) BiS, and (***C***) fEI for 59 iRBD patients at baseline (blue) and 46 healthy controls (gray). Shaded areas in the top row represent confidence intervals at 5% around the population mean (bootstrap, *n* = 1,000). The black shaded area in the bottom row indicated Cohen's *d* > 0.5 (i.e., moderate effect size). Group-level averaged of (***D***) DFA exponent, (***E***) BiS, and (***F***) fEI for 31 iRBD patients at baseline (blue) and at follow-up (red). Shaded areas represent confidence intervals at 5% around the population mean (bootstrap, *n* = 1,000). Generalized linear model results are summarized in Extended Data Figures 3-1–3-6.

Comparing the resting-state data of the two cohorts at baseline, we found (Fig. 3*A*,*B*; Extended Data Figs. 3-1, 3-2) that DFA scaling exponent and BiS were significantly stronger in iRBD patients than those in HC subjects in the delta (2–4 Hz) band (*p*_dfa_ = 0.0001 and *p*_bis_ = 0.0000001, Bonferroni *α* = 0.01) and theta (5–7 Hz) frequency band (*p*_dfa_ = 0.006 and *p*_bis_ = 0.014, Bonferroni *α* = 0.01). In addition, neuronal bistability was higher in iRBD patients than that in HC in the alpha (8–13 Hz) frequency band (*p*_bis_ = 0.027, Bonferroni *α* = 0.01). In contrast, iRBD patients showed (Fig. 3*C*, Extended Data Fig. 3-3) a significantly higher fEI in the delta (2–4 Hz) frequency band than HC subjects (*p*_fEI_ = 0.009, Bonferroni *α* = 0.01).

10.1523/JNEUROSCI.1871-24.2025.f3-1 Figure 3-1 Generalized linear model results for Detrended Fluctuation Analysis (DFA) scaling exponent, comparing healthy subjects and iRBD patients at baseline. Download Figure 3-1, DOCX file.

10.1523/JNEUROSCI.1871-24.2025.f3-2 Figure 3-2 Generalized linear model results for Bistability index (BiS), comparing healthy subjects and iRBD patients at baseline. Download Figure 3-2, DOCX file.

10.1523/JNEUROSCI.1871-24.2025.f3-3 Figure 3-3 Generalized linear model results for functional excitation-inhibition ratio (fEI), comparing healthy subjects and iRBD patients at baseline. Download Figure 3-3, DOCX file.

In the 31 iRBD patients who underwent EEG recording in two following time points, BiS in the alpha (8–13 Hz) band significantly decreased (Coef = −0.44, *p* = 0.006, Bonferroni *α* = 0.01) from baseline to follow-up (Fig. 3*E*, Extended Data Fig. 3-5). In addition, BiS decreased from baseline to follow-up in the theta (5–7 Hz, Coef = −0.45, *p* = 0.047) and in the beta (15–30 Hz, Coef = −0.44, *p* = 0.047) bands (Fig. 3*E*, Extended Data Fig. 3-5). DFA scaling exponents tended to change in the delta (Coef = −0.59, *p* = 0.022), in the alpha (Coef = −0.47, *p* = 0.045), and in the beta (Coef = −0.52, *p* = 0.021) bands (Fig. 3*D*, Extended Data Fig. 3-4).

**Figure 4.**
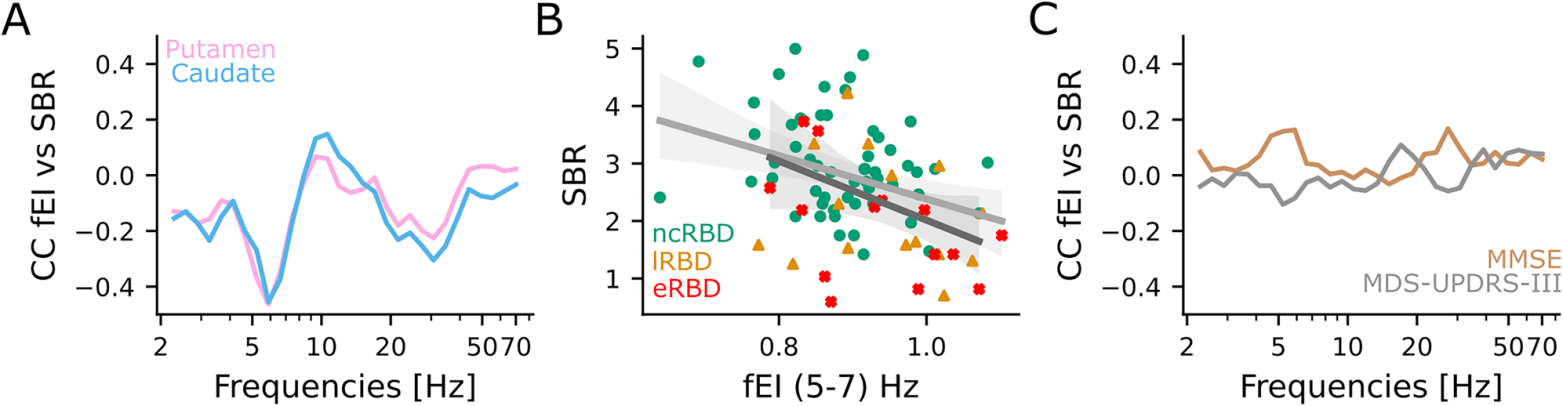
Hyperexcitability correlates with nigrostriatal dopaminergic impairment. ***A***, Spearman's correlation coefficient of averaged fEI across sensors with DaT levels in the putamen (pink) and caudate (azure). ***B***, The DaT levels in the putamen as a function of the theta (5–7 Hz) band fEI. Green circles, non-converters (*n* = 41 baseline recording, *n* = 17 instrumental follow-up recording); orange triangles, late-converters (*n* = 10 baseline recording, *n* = 6 instrumental follow-up recording); and red crosses, early-converters (*n* = 8 baseline recording, *n* = 8 instrumental follow-up recording). Continuous lines and shaded areas: average and confidence intervals of the linear regression model fit at baseline (light gray) and at follow-up (dark gray). ***C***, Kendall's correlation coefficient of averaged fEI across sensors with MMSE (brown) and MDS-UPDRS-III (gray). Linear mixed model results are summarized in Extended Data Figures 4-1, 4-2.

10.1523/JNEUROSCI.1871-24.2025.f3-4 Figure 3-4 Generalized linear model results for Detrended Fluctuation Analysis (DFA) scaling exponent, comparing iRBD patients at baseline and at follow-up. Download Figure 3-4, DOCX file.

**Figure 5.**
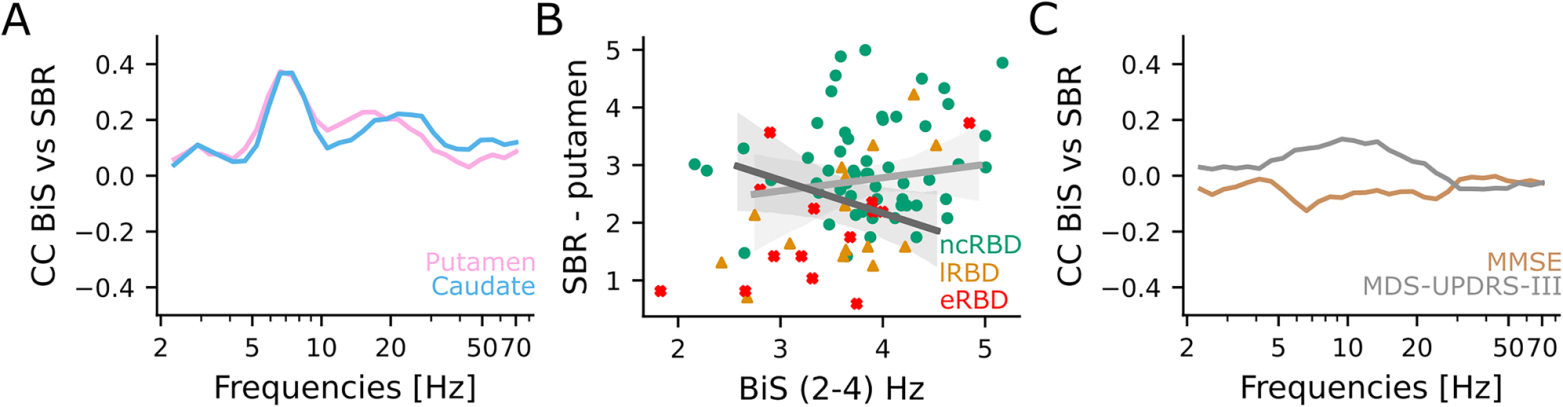
Mild neuronal bistability correlates with nigrostriatal dopaminergic impairment. ***A***, Spearman's correlation coefficient of averaged BiS across sensors with DaT levels in putamen (pink) and caudate (azure). ***B***, The DaT levels in the putamen as a function of delta (2–4 Hz) band BiS. Green circles, non-converters (*n* = 41 baseline recording, *n* = 17 instrumental follow-up recording); orange triangles, late-converters (*n* = 10 baseline recording, *n* = 6 instrumental follow-up recording); and red crosses, early-converters (*n* = 8 baseline recording, *n* = 8 instrumental follow-up recording). Continuous lines and shaded areas: average and confidence intervals of the linear regression model fit at baseline (light gray) and at follow-up (dark gray). ***C***, Kendall's correlation coefficient of averaged BiS across sensors with MMSE (brown) and MDS-UPDRS-III (gray). Linear mixed model results are summarized in Extended Data Figures 5-1, 5-2.

10.1523/JNEUROSCI.1871-24.2025.f3-5 Figure 3-5 Generalized linear model results for Bistability index (BiS), comparing iRBD patients at baseline and at follow-up. Download Figure 3-5, DOCX file.

**Figure 6.**
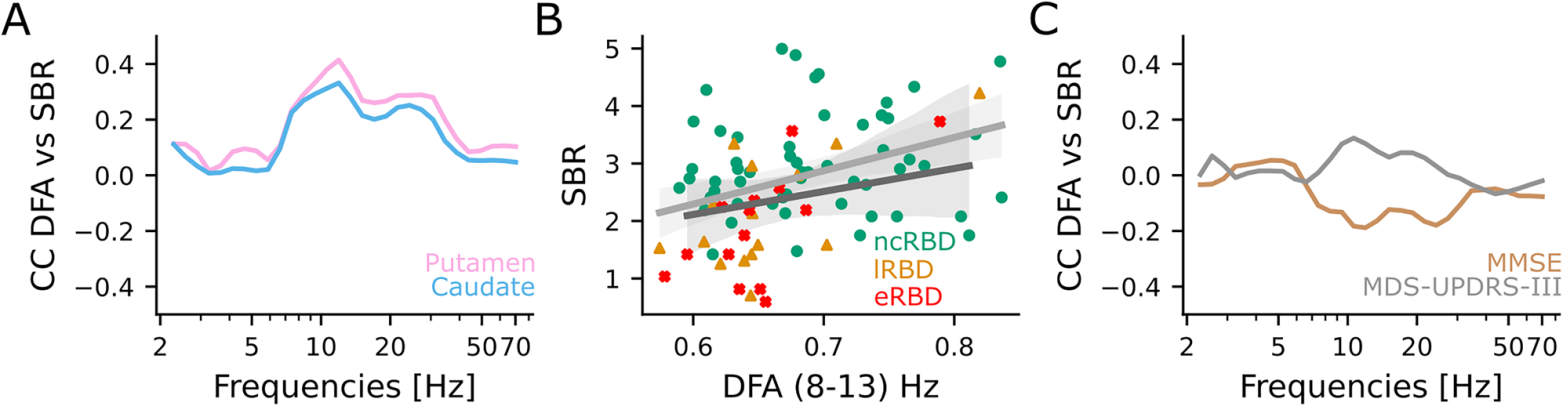
LRTCs positively correlate with nigrostriatal dopaminergic function. ***A***, Spearman's correlation coefficient of averaged DFA across sensors with DaT levels in putamen (pink) and caudate (azure). ***B***, The DaT levels in the putamen as a function of alpha (8–13 Hz) band DFA. Green circles, non-converters (*n* = 41 baseline recording, *n* = 17 instrumental follow-up recording); orange triangles, late-converters (*n* = 10 baseline recording, *n* = 6 instrumental follow-up recording); and red crosses, early-converters (*n* = 8 baseline recording, *n* = 8 instrumental follow-up recording). Continuous lines and shaded areas: average and confidence intervals of the linear regression model fit at baseline (light gray) and at follow-up (dark gray). ***C***, Kendall's correlation coefficient of averaged DFA across sensors with MMSE (brown) and MDS-UPDRS-III (gray). Linear mixed model results are summarized in Extended Data Figures 6-1, 6-2.

10.1523/JNEUROSCI.1871-24.2025.f3-6 Figure 3-6 Generalized linear model results for functional excitation-inhibition ratio (fEI), comparing iRBD patients at baseline and at follow-up. Download Figure 3-6, DOCX file.

In summary, iRBD patients showed at baseline altered cortical dynamics compared with HC mainly in the delta and theta frequency bands, and their operating point was shifted closer to the critical point (larger DFA) but still in an inhibition-dominated state (fEI < 1).

### Mild bistability and hyperexcitability are associated with nigrostriatal dopaminergic impairment

Nigrostriatal dopaminergic damage is associated with motor dysfunction and represents a conventional marker for iRBD patients with a more rapid clinical decline (Arnaldi et al., 2024). To investigate the relationship between nigrostriatal dopaminergic impairment with critical dynamics and E/I balance, we trained a LMM to predict the DaT levels (expressed by the SBRs obtained from DaT-SPECT) using fEI in canonical frequency bands. The DaT levels were negatively correlated (Coef = −0.260, *p* = 0.04) with fEI in the theta (5–7 Hz) band (Fig. 4*A*,*B*; Extended Data Fig. 4-1). Moreover, we suggested that early-converter iRBD patients (red crosses) and late-converter iRBD patients (orange triangles) exhibited higher values of fEI in the theta (5–7 Hz) band and lower levels of DaT than non-converter iRBD patients (Fig. 4*B*). We found no significant correlation between E/I balance and cognitive decline and motor impairment (Fig. 4*C*, Extended Data Fig. 4-2).

10.1523/JNEUROSCI.1871-24.2025.f4-1 Figure 4-1 Summary of the Linear Mixed Model (LMM) using as dependent variable the SBR in putamen, as fixed effect the fEI in canonical frequency bands, age, and sex, and as random effect subjects. Download Figure 4-1, DOCX file.

10.1523/JNEUROSCI.1871-24.2025.f4-2 Figure 4-2 Summary of the Linear Mixed Model (LMM) using as dependent variable clinical scores (i.e., MMSE or MDS-UPDRS-III), as fixed effect the fEI in canonical frequency bands, age, and sex, and as random effect subjects**.** Download Figure 4-2, DOCX file.

Subsequently, we investigated whether BiS predicts DaT levels and clinical score (Fig. 5). We found that DaT levels in putamen were significantly correlated with BiS in the delta (2–4 Hz, Coef = −0.31, *p* = 0.004) and in the theta (5–7 Hz, Coef = 0.29, *p* = 0.021) band (Fig. 5*A*,*B*; Extended Data Fig. 5-1). We suggested that early-converter (red crosses) and late-converter iRBD patients (orange triangles) showed higher values of BiS and lower levels of DaT in the delta (2–4 Hz) band than non-converter iRBD patients (Fig. 5*B*). Moreover, we did not observe any significant correlation between BiS and MDS-UPDRS-III or MMSE (Fig. 5*C*, Extended Data Fig. 5-2).

10.1523/JNEUROSCI.1871-24.2025.f5-1 Figure 5-1 Summary of the Linear Mixed Model (LMM) using as dependent variable the SBR in putamen, as fixed effect the BiS in canonical frequency bands, age, and sex, and as random effect subjects. Download Figure 5-1, DOCX file.

10.1523/JNEUROSCI.1871-24.2025.f5-2 Figure 5-2 Summary of the Linear Mixed Model (LMM) using as dependent variable clinical scores (i.e., MMSE or MDS-UPDRS-III), as fixed effect the BiS in canonical frequency bands, age, and sex, and as random effect subjects. Download Figure 5-2, DOCX file.

Finally, we investigated whether DFA scaling exponents predict DaT levels and clinical scores. DaT levels in putamen tended to correlate (Coef = 0.26, *p* = 0.11) with the DFA scaling exponent in the alpha (8–13 Hz) band (Fig. 6*A*, Extended Data Fig. 6-1). Notably, early-converter iRBD patients exhibited low values of DFA exponent correlated with low DaT levels (Fig. 6*B*). DFA exponents and clinical scores did not show any significant correlation (Fig. 6*C*, Extended Data Fig. 6-2).

10.1523/JNEUROSCI.1871-24.2025.f6-1 Figure 6-1 Summary of the Linear Mixed Model (LMM) using as dependent variable the SBR in putamen, as fixed effect the BiS in canonical frequency bands, age, and sex, and as random effect subjects. Download Figure 6-1, DOCX file.

10.1523/JNEUROSCI.1871-24.2025.f6-2 Figure 6-2 Summary of the Linear Mixed Model (LMM) using as dependent variable clinical scores (i.e., MMSE or MDS-UPDRS-III), as fixed effect the DFA in canonical frequency bands, age, and sex, and as random effect subjects. Download Figure 6-2, DOCX file.

Altogether these results show that cortical excitability, LRTCs, and neuronal bistability are correlated with significant nigrostriatal dopaminergic deafferentation.

### High synchronization correlates with hyperexcitability

**Figure 7.**
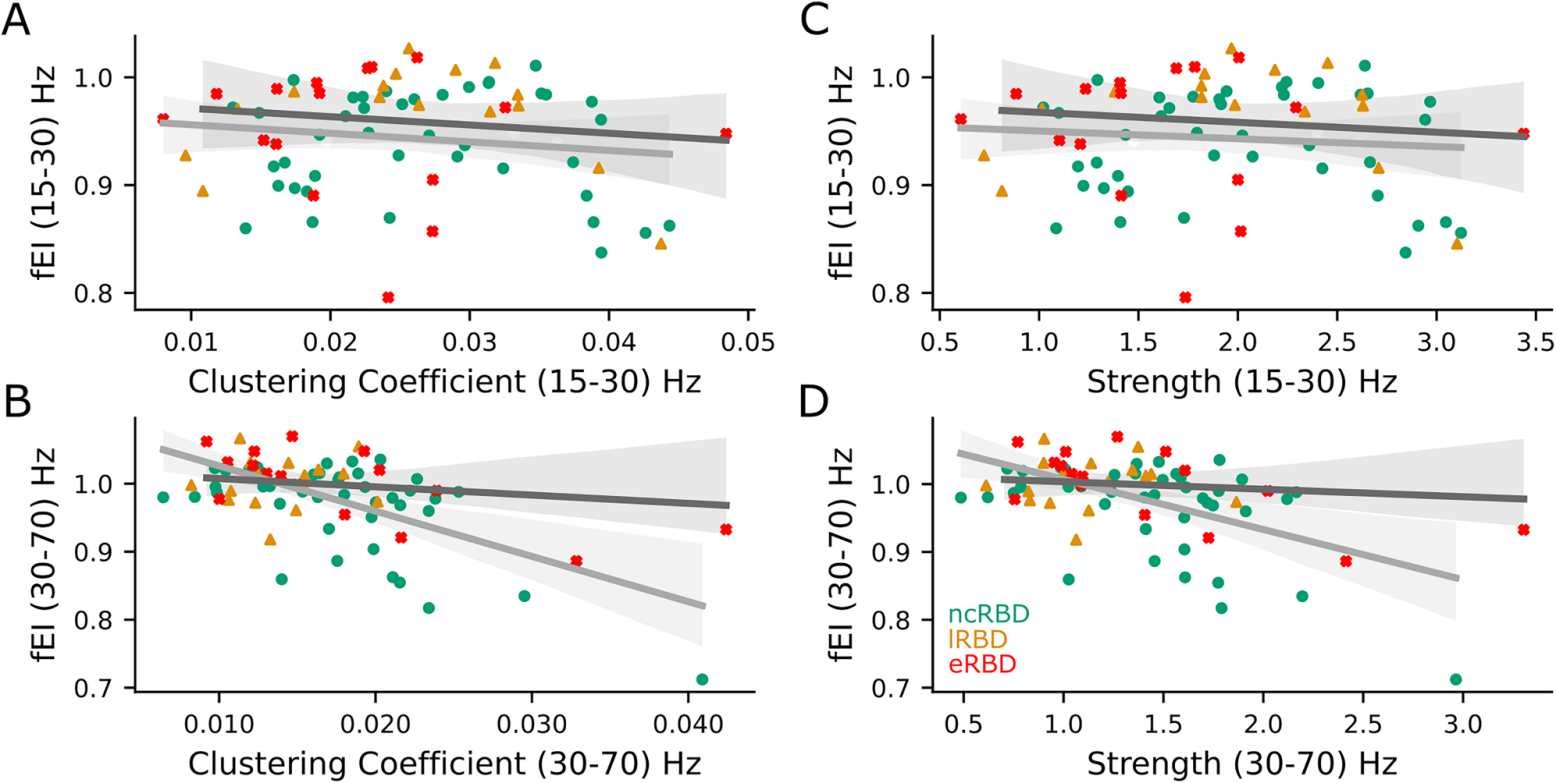
High phase synchronization correlates with hyperexcitability. ***A***, ***B***, Scatterplot of clustering coefficient and fEI in (***A***) beta (15–30 Hz) and (***B***) gamma (30–70 Hz) band. ***C***, ***D***, Scatterplot of strength and fEI in (***C***) beta band (15–30 Hz) and (***D***) gamma band (30–70 Hz). ***A–D***, Green circles show non-converter iRBD patients (*n* = 41 baseline recording, *n* = 17 follow-up recording), orange triangles show late-converter iRBD (*n* = 10 baseline recording, *n* = 6 follow-up recording), and red crosses show early-converter iRBD patients (*n* = 8 baseline recording, *n* = 8 follow-up recording). Linear mixed model results are summarized in Extended Data Figure 7-1.

Since iRBD increases the global phase synchronization, and this increase continues with the progression of the disease, we investigated the relationship between local E/I balance and nodal properties of interareal phase synchronization networks. Local E/I balance correlated with node strength and clustering coefficient (Fig. 7, Extended Data Fig. 7-1). In particular, the clustering coefficient was associated with fEI in the beta (15–30 Hz, Coef = −1.71, *p* = 0.031, Bonferroni *α* = 0.01) and gamma (30–70 Hz, Coef = −2.53, *p* = 0.000002, Bonferroni *α* = 0.01) bands (Fig. 7*A*,*C*). Nodal strength with fEI in the beta (15–30 Hz, Coef = 1.62, *p* = 0.040, Bonferroni *α* = 0.01) and gamma (30–70 Hz, Coef = 2.06, *p* = 0.0001, Bonferroni *α* = 0.01) bands (Fig. 7*B*,*D*).

10.1523/JNEUROSCI.1871-24.2025.f7-1 Figure 7-1 Summary of the five Linear Mixed Model (LMM) using as dependent variable the fEi in a frequency band (i.e., delta, theta, alpha, beta, and gamma), as fixed effect the nodal wPLI measures (i.e., strength, clustering coefficient, and eigen vector centrality) in each canonical frequency bands, age, and sex, and as random effect subjects. Download Figure 7-1, DOCX file.

## Discussion

By longitudinally evaluating a group of patients with iRBD, we sought to understand how critical cortical dynamics change from the prodromal phase of alpha-synucleinopathies to the overt stage of the disease. To this aim, we analyzed the high-density EEG data of 59 iRBD patients and 46 age-matched HC subjects by applying advanced quantitative analysis to extract several EEG features reflecting brain oscillations, under the conceptual framework of the critical brain hypothesis (Chialvo, 2010; Zimmern, 2020).

We found that spontaneous activity, mainly in the delta (2–4 Hz) and in the theta (5–7 Hz) frequency bands, of iRBD subjects exhibited higher bistability, higher long-range temporal correlations, and a smaller E/I ratio compared with age-matched healthy controls. These findings suggest that iRBD affects critical cortical dynamics before it is typically diagnosed, resulting in a shift of the brain's operating point toward supercriticality, with high levels of bistability.

As a second step, we evaluated the changes in brain oscillation metrics over time in a subgroup of enrolled iRBD patients with follow-up information, to gain insight into the longitudinal critical cortical dynamic modifications in patients with alpha-synucleinopathies, since the prodromal phase. Interestingly, bistability in the alpha band significantly decreases over time in our samples. LRTCs tended to decrease over time without surviving correction for multiple comparisons. This result suggests that at least some of the critical cortical dynamic modifications are an early event in the neurodegeneration cascade. However, given the small number of phenoconverted patients, it is possible that potential effects could not be observed because of the limited statistical power of our analysis.

Subsequently, we investigated the association between the critical cortical dynamic modifications and the biological and clinical severity of the disease. The EEG-derived criticality assessments were significantly associated with nigrostriatal dopaminergic function. Despite the absence of significant modulation of critical cortical dynamics in the longitudinal evaluation, the correlation between hyperexcitability and nigrostriatal dopaminergic impairment suggests that the critical cortical dynamics of iRBD reflect the worsening of the biological disease severity. It is interesting to highlight that the EEG-derived metrics only tended to be associated with clinical disease severity, but there is no significance, possibly for a floor effect. Idiopathic RBD patients may show only mild motor and cognitive symptoms for several years before phenoconversion or even appear entirely asymptomatic (Joza et al., 2023). On the other hand, the nigrostriatal dopaminergic pathway may be altered several years before phenoconversion (Arnaldi et al., 2021), even in the absence of overt clinical symptoms (Iranzo et al., 2017). Thus, the biological damage caused by the degeneration may be already detectable in cortical (EEG-derived criticality measures) and subcortical (nigrostriatal dopaminergic function) structures, without a concomitant clear clinical impairment.

Notably, we observed that early- and late-converter iRBD patients showed reduced LRTCs and bistability and increased excitability compared with non-converter iRBD patients. Importantly, it has been shown that individual LRTC exponents and levels of phase synchronization are correlated positively (Fuscà et al., 2023) in subcritical, but negatively in supercritical brain dynamics. Higher synchronization and hyperexcitability are both hallmarks of pathological conditions like epilepsy (Monto et al., 2007; Fuscà et al., 2023) and neurodegenerative diseases (Montez et al., 2009; Pusil et al., 2019; Maestú et al., 2021; Javed et al., 2022). Considering that iRBD patients show increased global phase synchronization with disease progression (Roascio et al., 2022) and that hyperexcitability is associated with a high neurodegeneration severity level, which characterized iRBD patients closer to phenoconversion, we suggest that the brain operating point shifts toward a supercritical regime with increased neurodegeneration severity.

In the present study, we observed that cortical hyperexcitation is associated with nigrostriatal dopaminergic impairment. Hence, we hypothesized that high phase synchronization and hyperexcitability, both of which are characteristic of iRBD patients closer to phenoconversion, were correlated in these patients. In the presented data, we found a strong correlation between high phase synchronization and hyperexcitability, both hallmarks of brain disorders (Sunwoo et al., 2017; Pusil et al., 2019; Javed et al., 2022), further suggesting a transition toward a supercritical regime. Therefore, even if no significant changes were demonstrated between baseline and follow-up assessments in our sample (perhaps due to the limited number of subjects), altogether these results suggest that once iRBD patients phenoconvert to the overt stage of the disease (i.e., by expressing parkinsonism and/or dementia), they exhibit oscillatory dynamics of a system closer to the “tip” (or center) of the critical regime. Thus, the phenoconversion seems to change cortical dynamics by further generating imbalance toward a hyperexcited state common to other brain disorders (Montez et al., 2009; Javed et al., 2022; van Nifterick et al., 2023).

Altogether these results extended reports of altered critical dynamics of the human brain from known effects in epilepsy (Linkenkaer-Hansen et al., 2005), autism spectrum disorders (Bruining et al., 2020), and Alzheimer's disease (Montez et al., 2009) since prodromal stages (Javed et al., 2022), also to prodromal stages of alpha-synucleinopathies. More intriguing, these findings support the hypothesis of early involvement of cortical and not only subcortical structures in the prodromal stage of alpha-synucleinopathy. Most disease models of Lewy body disorders suggest a bottom-up propagation of the alpha-synuclein-related pathology, where the cortical involvement is supposed to happen only in more advanced stages (Borghammer, 2023). The synuclein, origin, and connectome (SOC) disease model foresees a brain-first phenotype, where cortical structures may be involved earlier (Borghammer, 2023). However, iRBD patients should belong to the body-first phenotype, according to the SOC model. Conversely, our results suggest that neurodegeneration early affects cortical function in iRBD patients. This is in line with literature data showing cortical impairment in iRBD patients, detectable by both MRI (Churchill et al., 2024; Varga et al., 2024) and FDG-PET (Meles et al., 2018; Mattioli et al., 2023).

We acknowledge that this work has some limitations. First, instrumental follow-up time was not fixed but ranged from 12 to 36 months approximately. Moreover, not all patients underwent instrumental (i.e., hdEEG and DaT-SPECT) follow-up. It has to be highlighted that the enrolled patients are currently still being followed. Thus, future studies on this sample would include longer follow-ups in all patients, hopefully with more time points. This will allow more accurate modeling of clinical and instrumental longitudinal changes. Moreover, this is a single-center study, with a limited number of subjects. Considering that longitudinal data from both clinical and advanced techniques are not easy to collect, future multicenter studies with larger samples are needed to confirm the presented results. Furthermore, clinical longitudinal changes were assessed by using two metrics only (i.e., MMSE and MDS-UPDRS-III). Those two tools are commonly used as global measures of cognitive and motor function. However, it would be interesting to also evaluate the association between cortical dynamics alteration and more comprehensive cognitive and motor function biomarkers, as well as with other signs and symptoms usually present in alpha-synucleinopathies, such as autonomic function biomarkers. Antiepileptic drugs seem to have an inhibitory effect on cortical tissue (Meisel, 2020). It should be highlighted that iRBD patients may have started sleep medications, including clonazepam at the time of the follow-up visit, aimed at improving their sleep quality and possibly restoring the E/I balance. However, we observed no significant difference in E/I balance from baseline to follow-up, suggesting that drug administration does not necessarily affect critical dynamics in these patients. Thus, we could speculate that the hyperexcitability observed in the converted/progressive patients would be ascribed to real pathological effects rather than simpler administration of antiepileptic drugs.

In conclusion, this work shows that patients in the prodromal stage of alpha-synucleinopathies show cortical dynamic modifications by shifting the working point to excitation-dominated states getting closer to the overt stages of the disease. These results provide further evidence that both cortical and subcortical brain structures begin to be abnormal several years before the clinically overt syndrome (i.e., parkinsonism and/or dementia; Ferri et al., 2017). These results, on the one hand, increase our knowledge of the pathophysiological process underlying alpha-synucleinopathies since the prodromal stages. On the other hand, a better understanding of longitudinal cortical and subcortical modification along the continuum of alpha-synucleinopathies may provide new clues for innovative disease-modifying strategies, involving cerebral cortical structures.

## Data Availability

The data used in this work can be available upon a reasonable request due to privacy issues of clinical data. The Python codes are available at https://github.com/MonicaRoascio/RBD-criticality.git.

## References

[B1] American Academy of Sleep Medicine (2014) International classification of sleep disorder, Ed 3. Darien: American Academy of Sleep Medicine.

[B2] Arnaldi D, et al. (2021) Dopaminergic imaging and clinical predictors for phenoconversion of REM sleep behaviour disorder. Brain 144:278–287. 10.1093/brain/awaa365 33348363 PMC8599912

[B3] Arnaldi D, et al. (2024) Presynaptic dopaminergic imaging characterizes patients with REM sleep behavior disorder due to synucleinopathy. Ann Neurol 95:1178–1192. 10.1002/ana.26902 38466158 PMC11102309

[B4] Beggs JM (2008) The criticality hypothesis: how local cortical networks might optimize information processing. Philos Trans A Math Phys Eng Sci 366:329–343. 10.1098/rsta.2007.2092 17673410

[B5] Borghammer P (2023) The brain-first vs. body-first model of Parkinson’s disease with comparison to alternative models. J Neural Transm 130:737–753. 10.1007/s00702-023-02633-6 37062013

[B6] Braak H, Del Tredici K, Rüb U, De Vos RAI, Jansen Steur ENH, Braak E (2003) Staging of brain pathology related to sporadic Parkinson’s disease. Neurobiol Aging 24:197–211. 10.1016/S0197-4580(02)00065-9 12498954

[B7] Bruining H, et al. (2020) Measurement of excitation-inhibition ratio in autism spectrum disorder using critical brain dynamics. Sci Rep 10:9195. 10.1038/s41598-020-65500-4 32513931 PMC7280527

[B8] Burlando G, et al. (2025) Sleep-modulated cross-frequency coupling between δ phase and β-γ bistability: a system-level modulation of epileptic activity.

[B9] Chialvo DR (2010) Emergent complex neural dynamics. Nat Phys 6:744–750. 10.1038/nphys1803

[B10] Churchill L, Chen Y, Lewis SJG, Matar E (2024) Understanding REM sleep behavior disorder through functional MRI: a systematic review. Mov Disord 39:1679–1696. 10.1002/mds.29898 38934216

[B11] Cohen J (1988) Statistical power analysis for the behavioural science, Ed 2. New York: Routledge.

[B12] Darcourt J, Booij J, Tatsch K, Varrone A, Vander Borght T, Kapucu ÖL, Någren K, Nobili F, Walker Z, Van Laere K (2010) EANM procedure guidelines for brain neurotransmission SPECT using 123I-labelled dopamine transporter ligands, version 2. Eur J Nucl Med Mol Imaging 37:443–450. 10.1007/s00259-009-1267-x 19838702

[B13] di Santo S, Villegas P, Burioni R, Muñoz MA (2018) Landau–Ginzburg theory of cortex dynamics: scale-free avalanches emerge at the edge of synchronization. Proc Natl Acad Sci U S A 115:E1356–E1365. 10.1073/pnas.1712989115 29378970 PMC5816155

[B14] Fantini ML, Gagnon JF, Petit D, Rompré S, Décary A, Carrier J, Montplaisir J (2003) Slowing of electroencephalogram in rapid eye movement sleep behavior disorder. Ann Neurol 53:774–780. 10.1002/ana.10547 12783424

[B15] Ferri R, Rundo F, Silvani A, Zucconi M, Bruni O, Ferini-Strambi L, Plazzi G, Manconi M (2017) Rem sleep EEG instability in rem sleep behavior disorder and clonazepam effects. Sleep 40. 10.1093/sleep/zsx080 28482056

[B16] Freyer F, Aquino K, Robinson PA, Ritter P, Breakspear M (2009) Bistability and non-Gaussian fluctuations in spontaneous cortical activity. J Neurosci 29:8512–8524. 10.1523/JNEUROSCI.0754-09.2009 19571142 PMC6665653

[B17] Freyer F, Roberts JA, Becker R, Robinson PA, Ritter P, Breakspear M (2011) Biophysical mechanisms of multistability in resting-state cortical rhythms. J Neurosci 31:6353–6361. 10.1523/JNEUROSCI.6693-10.2011 21525275 PMC6622680

[B18] Freyer F, Roberts JA, Ritter P, Breakspear M (2012) A canonical model of multistability and scale-invariance in biological systems. PLoS Comput Biol 8. 10.1371/journal.pcbi.1002634 PMC3415415 22912567

[B19] Fuscà M, Siebenhühner F, Wang SH, Myrov V, Arnulfo G, Nobili L, Palva JM, Palva S (2023) Brain criticality predicts individual levels of inter-areal synchronization in human electrophysiological data. Nat Commun 14:2011–2022. 10.1038/s41467-023-40056-9 37550300 PMC10406818

[B20] Galbiati A, Verga L, Giora E, Zucconi M, Ferini-Strambi L (2019) The risk of neurodegeneration in REM sleep behavior disorder: a systematic review and meta-analysis of longitudinal studies. Sleep Med Rev 43:37–46. 10.1016/j.smrv.2018.09.008 30503716

[B21] Gilman S, et al. (2008) Second consensus statement on the diagnosis of multiple system atrophy. Neurology 71:670–676. 10.1212/01.wnl.0000324625.00404.15 18725592 PMC2676993

[B22] Hardstone R, Poil SS, Schiavone G, Jansen R, Nikulin VV, Mansvelder HD, Linkenkaer-Hansen K (2012) Detrended fluctuation analysis: a scale-free view on neuronal oscillations. Front Physiol 3:450. 10.3389/fphys.2012.00450 23226132 PMC3510427

[B23] Heinzel S, Berg D, Gasser T, Chen H, Yao C, Postuma RB (2019) Update of the MDS research criteria for prodromal Parkinson’s disease. Mov Disord 34:1464–1470. 10.1002/mds.27802 31412427

[B24] Iranzo A, Isetta V, Molinuevo JL, Serradell M, Navajas D, Farre R, Santamaria J (2010) Electroencephalographic slowing heralds mild cognitive impairment in idiopathic REM sleep behavior disorder. Sleep Med 11:534–539. 10.1016/j.sleep.2010.03.006 20462792

[B25] Iranzo A, et al. (2017) Dopamine transporter imaging deficit predicts early transition to synucleinopathy in idiopathic rapid eye movement sleep behavior disorder. Ann Neurol 82:419–428. 10.1002/ana.25026 28833467

[B26] Javed E, Sáurez-Méndez I, Susi G, Verdejo-Román J, Palva JM, Maestú F, Palva S (2022) E/I unbalance and aberrant oscillation dynamics predict preclinical Alzheimer’s disease. BioRxiv. 10.1523/JNEUROSCI.0688-24.2024 PMC11867000 40011070

[B27] Joza S, et al. (2023) Progression of clinical markers in prodromal Parkinson’s disease and dementia with Lewy bodies: a multicentre study. Brain 146:3258–3272. 10.1093/brain/awad072 36881989

[B28] Kinouchi O, Copelli M (2006) Optimal dynamical range of excitable networks at criticality. Nat Phys 2:348–352. 10.1038/nphys289

[B29] Linkenkaer-Hansen K, Nikouline VV, Palva JM, Ilmoniemi RJ (2001) Long-range temporal correlations and scaling behavior in human brain oscillations. J Neurosci 21:1370–1377. 10.1523/jneurosci.21-04-01370.2001 11160408 PMC6762238

[B30] Linkenkaer-Hansen K, Monto S, Rytsälä H, Suominen K, Isometsä E, Kähkönen S (2005) Breakdown of long-range temporal correlations in theta oscillations in patients with major depressive disorder. J Neurosci 25:10131–10137. 10.1523/JNEUROSCI.3244-05.2005 16267220 PMC6725784

[B31] Maestú F, de Haan W, Busche MA, DeFelipe J (2021) Neuronal excitation/inhibition imbalance: core element of a translational perspective on Alzheimer pathophysiology. Ageing Res Rev 69:101372. 10.1016/j.arr.2021.101372 34029743

[B32] Mattioli P, et al. (2023) Derivation and validation of a phenoconversion-related pattern in idiopathic rapid eye movement behavior disorder. Mov Disord 38:57–67. 10.1002/mds.29236 36190111 PMC10092506

[B33] McKeith IG, et al. (2017) Diagnosis and management of dementia with Lewy bodies. Neurology 89:88–100. 10.1212/WNL.0000000000004058 28592453 PMC5496518

[B34] McKeith IG, et al. (2020) Research criteria for the diagnosis of prodromal dementia with Lewy bodies. Neurology 94:743–755. 10.1212/WNL.0000000000009323 32241955 PMC7274845

[B35] Meisel C (2020) Antiepileptic drugs induce subcritical dynamics in human cortical networks. Proc Natl Acad Sci U S A 117:11118–11125. 10.1073/pnas.2000148117 32358198 PMC7245074

[B36] Meles SK, et al. (2018) The metabolic pattern of idiopathic REM sleep behavior disorder reflects early-stage Parkinson disease. J Nucl Med 59:1437–1444. 10.2967/jnumed.117.202242 29476004

[B37] Miglis MG, et al. (2021) Biomarkers of conversion to α-synucleinopathy in isolated rapid-eye-movement sleep behaviour disorder. Lancet Neurol 20:671–684. 10.1016/S1474-4422(21)00176-9 34302789 PMC8600613

[B38] Montez T, et al. (2009) Altered temporal correlations in parietal alpha and prefrontal theta oscillations in early-stage Alzheimer disease. Proc Natl Acad Sci U S A 106:1614–1619. 10.1073/pnas.0811699106 19164579 PMC2635782

[B39] Monto S, Vanhatalo S, Holmes MD, Palva JM (2007) Epileptogenic neocortical networks are revealed by abnormal temporal dynamics in seizure-free subdural EEG. Cereb Cortex 17:1386–1393. 10.1093/cercor/bhl049 16908492

[B40] Morbelli S, et al. (2020) EANM practice guideline/SNMMI procedure standard for dopaminergic imaging in Parkinsonian syndromes 1.0. Eur J Nucl Med Mol Imaging 47:1885–1912. 10.1007/s00259-020-04817-8 32388612 PMC7300075

[B41] Nobili F, et al. (2013) Automatic semi-quantification of [123I]FP-CIT SPECT scans in healthy volunteers using BasGan version 2: results from the ENC-DAT database. Eur J Nucl Med Mol Imaging 40:565–573. 10.1007/s00259-012-2304-8 23232506

[B42] Palva JM, Wang SH, Palva S, Zhigalov A, Monto S, Brookes MJ, Schoffelen JM, Jerbi K (2018) Ghost interactions in MEG/EEG source space: a note of caution on inter-areal coupling measures. Neuroimage 173:632–643. 10.1016/j.neuroimage.2018.02.032 29477441

[B43] Perrin F, Pernier J, Bertrand O, Echallier JF (1989) Spherical splines for scalp potential and current density mapping. Electroencephalogr Clin Neurophysiol 72:184–187. 10.1016/0013-4694(89)90180-6 2464490

[B44] Postuma RB, Gagnon JF, Bertrand JA, Génier Marchand D, Montplaisir JY (2015) Parkinson risk in idiopathic REM sleep behavior disorder: preparing for neuroprotective trials. Neurology 84:1104–1113. 10.1212/WNL.0000000000001364 25681454 PMC4371408

[B45] Postuma RB, et al. (2019) Risk and predictors of dementia and parkinsonism in idiopathic REM sleep behaviour disorder: a multicentre study. Brain 142:744–759. 10.1093/brain/awz030 30789229 PMC6391615

[B46] Priesemann V, Valderrama M, Wibral M, Le Van Quyen M (2013) Neuronal avalanches differ from wakefulness to deep sleep - evidence from intracranial depth recordings in humans. PLoS Comput Biol 9:e1002985. 10.1371/journal.pcbi.1002985 23555220 PMC3605058

[B47] Pusil S, López ME, Cuesta P, Bruña R, Pereda E, Maestú F (2019) Hypersynchronization in mild cognitive impairment: the ‘X’ model. Brain 142:3936–3950. 10.1093/brain/awz320 31633176

[B48] Roascio M, et al. (2022) Phase and amplitude electroencephalography correlations change with disease progression in people with idiopathic rapid eye-movement sleep behavior disorder. Sleep 45:zsab232. 10.1093/sleep/zsab232 34551110 PMC8754497

[B49] Spillantini MG, Schmidt ML, Lee VM-Y, Trojanowski JQ, Jakes R, Goedert M (1997) α-Synuclein in Lewy bodies. Nature 388:839–840. 10.1038/42166 9278044

[B50] Sunwoo JS, et al. (2017) Altered functional connectivity in idiopathic rapid eye movement sleep behavior disorder: a resting-state EEG study. Sleep 40:zsx058. 10.1093/sleep/zsx058 28431177

[B51] Tallon-Baudry C, Bertrand O, Peronnet F, Pernier J (1998) Induced γ-band activity during the delay of a visual short-term memory task in humans. J Neurosci 18:4244–4254. 10.1523/jneurosci.18-11-04244.1998 9592102 PMC6792803

[B52] Torrence C, Compo GP (1998) A practical guide to wavelet analysis. Bull Am Meteorol Soc 79:61–78. 10.1175/1520-0477(1998)079<0061:APGTWA>2.0.CO;2

[B53] van Nifterick AM, Mulder D, Duineveld DJ, Diachenko M, Scheltens P, Stam CJ, van Kesteren RE, Linkenkaer-Hansen K, Hillebrand A, Gouw AA (2023) Resting-state oscillations reveal disturbed excitation–inhibition ratio in Alzheimer’s disease patients. Sci Rep 13:7419. 10.1038/s41598-023-33973-8 37150756 PMC10164744

[B54] Varga Z, Keller J, Robinson SD, Serranova T, Nepozitek J, Zogala D, Trnka J, Ruzicka E, Sonka K, Dusek P (2024) Whole brain pattern of iron accumulation in REM sleep behavior disorder. Hum Brain Mapp 45:e26675. 10.1002/hbm.26675 38590155 PMC11002348

[B55] Vinck M, Oostenveld R, Van Wingerden M, Battaglia F, Pennartz CMA (2011) An improved index of phase-synchronization for electrophysiological data in the presence of volume-conduction, noise and sample-size bias. Neuroimage 55:1548–1565. 10.1016/j.neuroimage.2011.01.055 21276857

[B56] Wang SH, Siebenhühner F, Arnulfo G, Myrov V, Nobili L, Breakspear M, Palva S, Palva JM (2023) Critical-like brain dynamics in a continuum from second-to first-order phase transition. J Neurosci 43:7642–7656. 10.1523/JNEUROSCI.1889-22.2023 37816599 PMC10634584

[B57] Wang SH, Arnulfo G, Nobili L, Myrov V, Ferrari P, Ciuciu P, Palva S, Palva JM (2024a) Neuronal synchrony and critical bistability: mechanistic biomarkers for localizing the epileptogenic network. Epilepsia 65:2041–2053. 10.1111/epi.17996 38687176

[B58] Wang SH, Marzulli M, Arnulfo G, Nobili L, Palva S, Palva JM, Ciuciu P (2024b) Machine learning models trained in a low-dimensional latent space for epileptogenic zone (EZ) localization. 2024 32nd European signal processing conference (EUSIPCO), 1586–1590.

[B59] Wilting J, Priesemann V (2019) 25 Years of criticality in neuroscience—established results, open controversies, novel concepts. Curr Opin Neurobiol 58:105–111. 10.1016/j.conb.2019.08.002 31546053

[B60] Zimmern V (2020) Why brain criticality is clinically relevant: a scoping review. Front Neural Circuits 14:54. 10.3389/fncir.2020.00054 32982698 PMC7479292

