# Supplementary material for: Unveiling Cortical Criticality Changes along the Prodromal to the Overt Continuum of Alpha-Synucleinopathy
Source: J Neurosci. 2025 Jul 3;45(31):e1871242025. doi: 10.1523/JNEUROSCI.1871-24.2025 (PMC12311758; doi:10.1523/JNEUROSCI.1871-24.2025)
Supplement: Figure 3-1 — Generalized linear model results for Detrended Fluctuation Analysis (DFA) scaling exponent, comparing healthy subjects and iRBD patients at baseline. Download Figure 3-1, DOCX file. [file jneuro-45-e1871242025-s001.docx]

**Figure 3-1**: Generalized linear model results for Detrended Fluctuation Analysis (DFA) scaling exponent, comparing healthy subjects and iRBD patients at baseline.

|  | **Coef.** | **Std.Err.** | **z** | **P>\|z\|** | **[0.025** | **0.975]** | **Dep. Var.** |
| --- | --- | --- | --- | --- | --- | --- | --- |
| **Intercept** | -0.308 | 0.777 | -0.397 | 0.6913 | -1.830 | 1.214 | DFA 2-4 Hz |
| **Groups[T.RBD]** | 0.747 | 0.192 | 3.884 | 0.0001 | 0.370 | 1.124 | DFA 2-4 Hz |
| **Sex[T.M]** | -0.221 | 0.212 | -1.043 | 0.2971 | -0.635 | 0.194 | DFA 2-4 Hz |
| **Age** | 0.003 | 0.011 | 0.296 | 0.7674 | -0.017 | 0.024 | DFA 2-4 Hz |
| **Intercept** | 0.671 | 0.787 | 0.853 | 0.3939 | -0.871 | 2.212 | DFA 5-7 Hz |
| **Groups[T.RBD]** | 0.530 | 0.195 | 2.717 | 0.0066 | 0.148 | 0.912 | DFA 5-7 Hz |
| **Sex[T.M]** | -0.343 | 0.214 | -1.599 | 0.1098 | -0.763 | 0.077 | DFA 5-7 Hz |
| **Age** | -0.009 | 0.011 | -0.801 | 0.4229 | -0.029 | 0.012 | DFA 5-7 Hz |
| **Intercept** | 1.096 | 0.595 | 1.841 | 0.0656 | -0.071 | 2.262 | DFA 8-13 Hz |
| **Groups[T.RBD]** | -0.090 | 0.147 | -0.608 | 0.5434 | -0.379 | 0.199 | DFA 8-13 Hz |
| **Sex[T.M]** | -0.021 | 0.162 | -0.129 | 0.8975 | -0.339 | 0.297 | DFA 8-13 Hz |
| **Age** | -0.013 | 0.008 | -1.658 | 0.0973 | -0.029 | 0.002 | DFA 8-13 Hz |
| **Intercept** | 0.679 | 0.585 | 1.161 | 0.2456 | -0.467 | 1.826 | DFA 15-30 Hz |
| **Groups[T.RBD]** | -0.129 | 0.145 | -0.889 | 0.3740 | -0.413 | 0.155 | DFA 15-30 Hz |
| **Sex[T.M]** | 0.075 | 0.159 | 0.468 | 0.6397 | -0.238 | 0.387 | DFA 15-30 Hz |
| **Age** | -0.008 | 0.008 | -0.992 | 0.3214 | -0.023 | 0.008 | DFA 15-30 Hz |
| **Intercept** | 0.722 | 0.628 | 1.150 | 0.2500 | -0.508 | 1.952 | DFA 30 -70 Hz |
| **Groups[T.RBD]** | -0.212 | 0.156 | -1.365 | 0.1723 | -0.517 | 0.093 | DFA 30 -70 Hz |
| **Sex[T.M]** | -0.046 | 0.171 | -0.272 | 0.7859 | -0.382 | 0.289 | DFA 30 -70 Hz |
| **Age** | -0.006 | 0.008 | -0.756 | 0.4498 | -0.023 | 0.010 | DFA 30 -70 Hz |
