# Supplementary material for: Unveiling Cortical Criticality Changes along the Prodromal to the Overt Continuum of Alpha-Synucleinopathy
Source: J Neurosci. 2025 Jul 3;45(31):e1871242025. doi: 10.1523/JNEUROSCI.1871-24.2025 (PMC12311758; doi:10.1523/JNEUROSCI.1871-24.2025)
Supplement: Figure 3-2 — Generalized linear model results for Bistability index (BiS), comparing healthy subjects and iRBD patients at baseline. Download Figure 3-2, DOCX file. [file jneuro-45-e1871242025-s002.docx]

**Figure 3-2:** Generalized linear model results for Bistability index (BiS), comparing healthy subjects and iRBD patients at baseline.

|  | **Coef.** | **Std.Err.** | **z** | **P>\|z\|** | **[0.025** | **0.975]** | **Dep. Var.** |
| --- | --- | --- | --- | --- | --- | --- | --- |
| **Intercept** | -0.708 | 0.749 | -0.945 | 0.3448386 | -2.176 | 0.761 | BiS 2-4Hz |
| **Groups[T.RBD]** | 0.994 | 0.186 | 5.356 | 0.0000001 | 0.631 | 1.358 | BiS 2-4Hz |
| **Sex[T.M]** | -0.462 | 0.204 | -2.265 | 0.0235431 | -0.863 | -0.062 | BiS 2-4Hz |
| **Age** | 0.006 | 0.010 | 0.550 | 0.5822482 | -0.014 | 0.025 | BiS 2-4Hz |
| **Intercept** | 1.401 | 0.729 | 1.922 | 0.0546641 | -0.028 | 2.830 | BiS 5-7 Hz |
| **Groups[T.RBD]** | 0.444 | 0.181 | 2.458 | 0.0139899 | 0.090 | 0.798 | BiS 5-7 Hz |
| **Sex[T.M]** | -0.633 | 0.199 | -3.187 | 0.0014376 | -1.023 | -0.244 | BiS 5-7 Hz |
| **Age** | -0.018 | 0.010 | -1.839 | 0.0659836 | -0.037 | 0.001 | BiS 5-7 Hz |
| **Intercept** | 2.447 | 0.612 | 4.000 | 0.0000633 | 1.248 | 3.646 | BiS 8-13 Hz |
| **Groups[T.RBD]** | 0.335 | 0.152 | 2.209 | 0.0271829 | 0.038 | 0.632 | BiS 8-13 Hz |
| **Sex[T.M]** | -0.611 | 0.167 | -3.663 | 0.0002498 | -0.937 | -0.284 | BiS 8-13 Hz |
| **Age** | -0.032 | 0.008 | -3.858 | 0.0001144 | -0.048 | -0.016 | BiS 8-13 Hz |
| **Intercept** | 0.800 | 0.675 | 1.186 | 0.2357174 | -0.523 | 2.123 | BiS 15-30 Hz |
| **Groups[T.RBD]** | 0.273 | 0.167 | 1.633 | 0.1024492 | -0.055 | 0.601 | BiS 15-30 Hz |
| **Sex[T.M]** | -0.458 | 0.184 | -2.487 | 0.0128699 | -0.818 | -0.097 | BiS 15-30 Hz |
| **Age** | -0.010 | 0.009 | -1.123 | 0.2613764 | -0.028 | 0.008 | BiS 15-30 Hz |
| **Intercept** | 0.209 | 0.748 | 0.279 | 0.7800605 | -1.257 | 1.674 | BiS 30-70 Hz |
| **Groups[T.RBD]** | 0.267 | 0.185 | 1.442 | 0.1494268 | -0.096 | 0.630 | BiS 30-70 Hz |
| **Sex[T.M]** | -0.373 | 0.204 | -1.832 | 0.0669343 | -0.773 | 0.026 | BiS 30-70 Hz |
| **Age** | -0.001 | 0.010 | -0.056 | 0.9557138 | -0.020 | 0.019 | BiS 30-70 Hz |
