# Supplementary material for: Unveiling Cortical Criticality Changes along the Prodromal to the Overt Continuum of Alpha-Synucleinopathy
Source: J Neurosci. 2025 Jul 3;45(31):e1871242025. doi: 10.1523/JNEUROSCI.1871-24.2025 (PMC12311758; doi:10.1523/JNEUROSCI.1871-24.2025)
Supplement: Figure 3-3 — Generalized linear model results for functional excitation-inhibition ratio (fEI), comparing healthy subjects and iRBD patients at baseline. Download Figure 3-3, DOCX file. [file jneuro-45-e1871242025-s003.docx]

**Figure 3-3:** Generalized linear model results for functional excitation-inhibition ratio (fEI), comparing healthy subjects and iRBD patients at baseline.

|  | **Coef.** | **Std.Err.** | **z** | **P>\|z\|** | **[0.025** | **0.975]** | **Dep. Var.** |
| --- | --- | --- | --- | --- | --- | --- | --- |
| **Intercept** | -0.328 | 0.778 | -0.422 | 0.673 | -1.853 | 1.196 | fEI 2-4Hz |
| **Groups[T.RBD]** | -0.505 | 0.193 | -2.621 | 0.009 | -0.883 | -0.127 | fEI 2-4Hz |
| **Sex[T.M]** | 0.311 | 0.212 | 1.466 | 0.143 | -0.105 | 0.726 | fEI 2-4Hz |
| **Age** | 0.003 | 0.011 | 0.255 | 0.799 | -0.018 | 0.023 | fEI 2-4Hz |
| **Intercept** | -2.535 | 0.614 | -4.128 | 0.000 | -3.739 | -1.331 | fEI 5-7 Hz |
| **Groups[T.RBD]** | 0.130 | 0.152 | 0.852 | 0.394 | -0.169 | 0.428 | fEI 5-7 Hz |
| **Sex[T.M]** | 0.454 | 0.167 | 2.712 | 0.007 | 0.126 | 0.782 | fEI 5-7 Hz |
| **Age** | 0.032 | 0.008 | 3.819 | 0.000 | 0.015 | 0.048 | fEI 5-7 Hz |
| **Intercept** | -1.716 | 0.658 | -2.609 | 0.009 | -3.005 | -0.427 | fEI 8-13 Hz |
| **Groups[T.RBD]** | 0.156 | 0.163 | 0.960 | 0.337 | -0.163 | 0.476 | fEI 8-13 Hz |
| **Sex[T.M]** | 0.156 | 0.179 | 0.871 | 0.384 | -0.195 | 0.507 | fEI 8-13 Hz |
| **Age** | 0.021 | 0.009 | 2.355 | 0.019 | 0.004 | 0.038 | fEI 8-13 Hz |
| **Intercept** | -2.526 | 0.741 | -3.407 | 0.001 | -3.979 | -1.073 | fEI 15-30 Hz |
| **Groups[T.RBD]** | -0.155 | 0.184 | -0.843 | 0.399 | -0.515 | 0.205 | fEI 15-30 Hz |
| **Sex[T.M]** | 0.221 | 0.202 | 1.094 | 0.274 | -0.175 | 0.617 | fEI 15-30 Hz |
| **Age** | 0.031 | 0.010 | 3.105 | 0.002 | 0.011 | 0.051 | fEI 15-30 Hz |
| **Intercept** | -1.644 | 1.261 | -1.303 | 0.192 | -4.116 | 0.828 | fEI 30-70 Hz |
| **Groups[T.RBD]** | -0.383 | 0.313 | -1.225 | 0.221 | -0.995 | 0.230 | fEI 30-70 Hz |
| **Sex[T.M]** | 0.492 | 0.344 | 1.431 | 0.152 | -0.182 | 1.166 | fEI 30-70 Hz |
| **Age** | 0.016 | 0.017 | 0.950 | 0.342 | -0.017 | 0.050 | fEI 30-70 Hz |
