# Supplementary material for: Unveiling Cortical Criticality Changes along the Prodromal to the Overt Continuum of Alpha-Synucleinopathy
Source: J Neurosci. 2025 Jul 3;45(31):e1871242025. doi: 10.1523/JNEUROSCI.1871-24.2025 (PMC12311758; doi:10.1523/JNEUROSCI.1871-24.2025)
Supplement: Figure 3-4 — Generalized linear model results for Detrended Fluctuation Analysis (DFA) scaling exponent, comparing iRBD patients at baseline and at follow-up. Download Figure 3-4, DOCX file. [file jneuro-45-e1871242025-s004.docx]

**Figure 3-4**: Generalized linear model results for Detrended Fluctuation Analysis (DFA) scaling exponent, comparing iRBD patients at baseline and at follow-up.

|  | **Coef.** | **Std.Err.** | **z** | **P>\|z\|** | **[0.025** | **0.975]** | **Dep. Var.** |
| --- | --- | --- | --- | --- | --- | --- | --- |
| **Intercept** | -1.635 | 1.374 | -1.190 | 0.234 | -4.328 | 1.059 | DFA 2-4 Hz |
| **Groups[T.FUP]** | -0.591 | 0.258 | -2.289 | 0.022 | -1.097 | -0.085 | DFA 2-4 Hz |
| **Sex[T.M]** | 0.028 | 0.019 | 1.461 | 0.144 | -0.010 | 0.067 | DFA 2-4 Hz |
| **Age** | 1.255 | 0.323 | 3.889 | 0.000 | 0.622 | 1.887 | DFA 2-4 Hz |
| **Intercept** | -1.327 | 1.530 | -0.867 | 0.386 | -4.325 | 1.672 | DFA 5-7 Hz |
| **Groups[T. FUP]** | -0.460 | 0.287 | -1.601 | 0.109 | -1.024 | 0.103 | DFA 5-7 Hz |
| **Sex[T.M]** | 0.022 | 0.022 | 1.023 | 0.306 | -0.020 | 0.065 | DFA 5-7 Hz |
| **Age** | 1.094 | 0.359 | 3.046 | 0.002 | 0.390 | 1.798 | DFA 5-7 Hz |
| **Intercept** | -0.049 | 1.252 | -0.039 | 0.969 | -2.503 | 2.405 | DFA 8-13 Hz |
| **Groups[T. FUP]** | -0.473 | 0.235 | -2.009 | 0.045 | -0.934 | -0.012 | DFA 8-13 Hz |
| **Sex[T.M]** | 0.005 | 0.018 | 0.297 | 0.766 | -0.030 | 0.040 | DFA 8-13 Hz |
| **Age** | 0.273 | 0.294 | 0.929 | 0.353 | -0.303 | 0.849 | DFA 8-13 Hz |
| **Intercept** | -1.242 | 1.200 | -1.035 | 0.300 | -3.593 | 1.109 | DFA 15-30 Hz |
| **Groups[T. FUP]** | -0.521 | 0.225 | -2.311 | 0.021 | -0.963 | -0.079 | DFA 15-30 Hz |
| **Sex[T.M]** | 0.024 | 0.017 | 1.389 | 0.165 | -0.010 | 0.057 | DFA 15-30 Hz |
| **Age** | -0.042 | 0.282 | -0.150 | 0.881 | -0.594 | 0.510 | DFA 15-30 Hz |
| **Intercept** | -0.036 | 1.069 | -0.034 | 0.973 | -2.132 | 2.059 | DFA 30 -70 Hz |
| **Groups[T. FUP]** | -0.127 | 0.201 | -0.633 | 0.527 | -0.521 | 0.267 | DFA 30 -70 Hz |
| **Sex[T.M]** | 0.002 | 0.015 | 0.130 | 0.896 | -0.028 | 0.032 | DFA 30 -70 Hz |
| **Age** | 0.051 | 0.251 | 0.202 | 0.840 | -0.441 | 0.543 | DFA 30 -70 Hz |
