# Supplementary material for: Unveiling Cortical Criticality Changes along the Prodromal to the Overt Continuum of Alpha-Synucleinopathy
Source: J Neurosci. 2025 Jul 3;45(31):e1871242025. doi: 10.1523/JNEUROSCI.1871-24.2025 (PMC12311758; doi:10.1523/JNEUROSCI.1871-24.2025)
Supplement: Figure 3-5 — Generalized linear model results for Bistability index (BiS), comparing iRBD patients at baseline and at follow-up. Download Figure 3-5, DOCX file. [file jneuro-45-e1871242025-s005.docx]

**Figure 3-5:** Generalized linear model results for Bistability index (BiS), comparing iRBD patients at baseline and at follow-up.

|  | **Coef.** | **Std.Err.** | **z** | **P>\|z\|** | **[0.025** | **0.975]** | **Dep. Var.** |
| --- | --- | --- | --- | --- | --- | --- | --- |
| **Intercept** | -1.574 | 1.403 | -1.122 | 0.262 | -4.323 | 1.175 | BiS 2-4Hz |
| **Groups[T. FUP]** | -0.464 | 0.264 | -1.760 | 0.078 | -0.980 | 0.053 | BiS 2-4Hz |
| **Sex[T.M]** | 0.023 | 0.020 | 1.136 | 0.256 | -0.016 | 0.062 | BiS 2-4Hz |
| **Age** | 1.132 | 0.329 | 3.437 | 0.001 | 0.487 | 1.778 | BiS 2-4Hz |
| **Intercept** | 0.171 | 1.214 | 0.141 | 0.888 | -2.209 | 2.551 | BiS 5-7 Hz |
| **Groups[T. FUP]** | -0.454 | 0.228 | -1.989 | 0.047 | -0.901 | -0.007 | BiS 5-7 Hz |
| **Sex[T.M]** | -0.003 | 0.017 | -0.185 | 0.853 | -0.037 | 0.031 | BiS 5-7 Hz |
| **Age** | 0.917 | 0.285 | 3.217 | 0.001 | 0.358 | 1.476 | BiS 5-7 Hz |
| **Intercept** | -0.849 | 0.846 | -1.004 | 0.316 | -2.507 | 0.809 | BiS 8-13 Hz |
| **Groups[T. FUP]** | -0.439 | 0.159 | -2.762 | 0.006 | -0.751 | -0.128 | BiS 8-13 Hz |
| **Sex[T.M]** | 0.015 | 0.012 | 1.237 | 0.216 | -0.009 | 0.038 | BiS 8-13 Hz |
| **Age** | 0.007 | 0.199 | 0.037 | 0.971 | -0.382 | 0.397 | BiS 8-13 Hz |
| **Intercept** | -1.505 | 1.032 | -1.458 | 0.145 | -3.529 | 0.518 | BiS 15-30 Hz |
| **Groups[T. FUP]** | -0.442 | 0.194 | -2.280 | 0.023 | -0.823 | -0.062 | BiS 15-30 Hz |
| **Sex[T.M]** | 0.022 | 0.015 | 1.502 | 0.133 | -0.007 | 0.051 | BiS 15-30 Hz |
| **Age** | 0.459 | 0.242 | 1.892 | 0.058 | -0.016 | 0.934 | BiS 15-30 Hz |
| **Intercept** | -0.743 | 1.105 | -0.673 | 0.501 | -2.909 | 1.422 | BiS 30-70 Hz |
| **Groups[T. FUP]** | -0.141 | 0.208 | -0.681 | 0.496 | -0.548 | 0.265 | BiS 30-70 Hz |
| **Sex[T.M]** | 0.008 | 0.016 | 0.496 | 0.620 | -0.023 | 0.038 | BiS 30-70 Hz |
| **Age** | 1.062 | 0.259 | 4.092 | 0.000 | 0.553 | 1.570 | BiS 30-70 Hz |
