# Supplementary material for: Unveiling Cortical Criticality Changes along the Prodromal to the Overt Continuum of Alpha-Synucleinopathy
Source: J Neurosci. 2025 Jul 3;45(31):e1871242025. doi: 10.1523/JNEUROSCI.1871-24.2025 (PMC12311758; doi:10.1523/JNEUROSCI.1871-24.2025)
Supplement: Figure 3-6 — Generalized linear model results for functional excitation-inhibition ratio (fEI), comparing iRBD patients at baseline and at follow-up. Download Figure 3-6, DOCX file. [file jneuro-45-e1871242025-s006.docx]

**Figure 3-6:** Generalized linear model results for functional excitation-inhibition ratio (fEI), comparing iRBD patients at baseline and at follow-up.

|  | **Coef.** | **Std.Err.** | **z** | **P>\|z\|** | **[0.025** | **0.975]** | **Dep. Var.** |
| --- | --- | --- | --- | --- | --- | --- | --- |
| **Intercept** | -1.032 | 1.147 | -0.900 | 0.368 | -3.280 | 1.216 | fEI 2-4Hz |
| **Groups[T.RBD]** | 0.184 | 0.216 | 0.853 | 0.393 | -0.239 | 0.607 | fEI 2-4Hz |
| **Sex[T.M]** | 0.013 | 0.016 | 0.826 | 0.409 | -0.018 | 0.045 | fEI 2-4Hz |
| **Age** | -0.386 | 0.269 | -1.432 | 0.152 | -0.914 | 0.142 | fEI 2-4Hz |
| **Intercept** | -1.288 | 0.888 | -1.451 | 0.147 | -3.028 | 0.452 | fEI 5-7 Hz |
| **Groups[T.RBD]** | 0.108 | 0.167 | 0.649 | 0.517 | -0.219 | 0.435 | fEI 5-7 Hz |
| **Sex[T.M]** | 0.017 | 0.013 | 1.388 | 0.165 | -0.007 | 0.042 | fEI 5-7 Hz |
| **Age** | 0.012 | 0.208 | 0.057 | 0.954 | -0.397 | 0.420 | fEI 5-7 Hz |
| **Intercept** | 1.115 | 0.962 | 1.159 | 0.247 | -0.771 | 3.001 | fEI 8-13 Hz |
| **Groups[T.RBD]** | 0.042 | 0.181 | 0.232 | 0.816 | -0.312 | 0.397 | fEI 8-13 Hz |
| **Sex[T.M]** | -0.019 | 0.014 | -1.410 | 0.158 | -0.046 | 0.008 | fEI 8-13 Hz |
| **Age** | 0.865 | 0.226 | 3.828 | 0.000 | 0.422 | 1.308 | fEI 8-13 Hz |
| **Intercept** | -1.462 | 1.219 | -1.199 | 0.230 | -3.852 | 0.928 | fEI 15-30 Hz |
| **Groups[T.RBD]** | 0.241 | 0.229 | 1.053 | 0.292 | -0.208 | 0.690 | fEI 15-30 Hz |
| **Sex[T.M]** | 0.012 | 0.017 | 0.713 | 0.476 | -0.022 | 0.046 | fEI 15-30 Hz |
| **Age** | 0.467 | 0.286 | 1.632 | 0.103 | -0.094 | 1.028 | fEI 15-30 Hz |
| **Intercept** | -2.813 | 1.084 | -2.596 | 0.009 | -4.937 | -0.689 | fEI 30-70 Hz |
| **Groups[T.RBD]** | 0.020 | 0.204 | 0.100 | 0.921 | -0.379 | 0.419 | fEI 30-70 Hz |
| **Sex[T.M]** | 0.039 | 0.015 | 2.553 | 0.011 | 0.009 | 0.069 | fEI 30-70 Hz |
| **Age** | -0.719 | 0.254 | -2.825 | 0.005 | -1.218 | -0.220 | fEI 30-70 Hz |
