# Supplementary material for: Unveiling Cortical Criticality Changes along the Prodromal to the Overt Continuum of Alpha-Synucleinopathy
Source: J Neurosci. 2025 Jul 3;45(31):e1871242025. doi: 10.1523/JNEUROSCI.1871-24.2025 (PMC12311758; doi:10.1523/JNEUROSCI.1871-24.2025)
Supplement: Figure 4-1 — Summary of the Linear Mixed Model (LMM) using as dependent variable the SBR in putamen, as fixed effect the fEI in canonical frequency bands, age, and sex, and as random effect subjects. Download Figure 4-1, DOCX file. [file jneuro-45-e1871242025-s007.docx]

**Figure 4-1**: Summary of the Linear Mixed Model (LMM) using as dependent variable the SBR in putamen, as fixed effect the fEI in canonical frequency bands, age, and sex, and as random effect the subjects.

|  | **Coef.** | **Std.Err.** | **z** | **P>\|z\|** | **[0.025** | **0.975]** |
| --- | --- | --- | --- | --- | --- | --- |
| **Intercept** | 2.963 | 1.359 | 2.181 | 0.029 | 0.300 | 5.626 |
| **Sex[T.M]** | -0.379 | 0.362 | -1.047 | 0.295 | -1.090 | 0.331 |
| **fEI 2-4 Hz** | 0.055 | 0.076 | 0.729 | 0.466 | -0.093 | 0.204 |
| **fEI 5-7 Hz** | -0.255 | 0.124 | -2.053 | 0.040 | -0.499 | -0.012 |
| **fEI 8-13 Hz** | 0.122 | 0.117 | 1.041 | 0.298 | -0.108 | 0.352 |
| **fEI 15-30 Hz** | -0.097 | 0.121 | -0.799 | 0.424 | -0.335 | 0.141 |
| **fEI 30-70 Hz** | 0.014 | 0.090 | 0.161 | 0.872 | -0.162 | 0.191 |
| **Age** | -0.037 | 0.018 | -2.084 | 0.037 | -0.072 | -0.002 |
| **Group Var** | 0.758 | 0.886 |  |  |  |  |
