# Supplementary material for: Unveiling Cortical Criticality Changes along the Prodromal to the Overt Continuum of Alpha-Synucleinopathy
Source: J Neurosci. 2025 Jul 3;45(31):e1871242025. doi: 10.1523/JNEUROSCI.1871-24.2025 (PMC12311758; doi:10.1523/JNEUROSCI.1871-24.2025)
Supplement: Figure 4-2 — Summary of the Linear Mixed Model (LMM) using as dependent variable clinical scores (i.e., MMSE or MDS-UPDRS-III), as fixed effect the fEI in canonical frequency bands, age, and sex, and as random effect subjects. Download Figure 4-2, DOCX file. [file jneuro-45-e1871242025-s008.docx]

**Figure 4-2**: Summary of the Linear Mixed Model (LMM) using as dependent variable clinical scores (i.e., MMSE or MDS-UPDRS-III), as fixed effect the fEI in canonical frequency bands, age, and sex, and as random effect the subjects.

|  | **Coef.** | **Std.Err.** | **z** | **P>\|z\|** | **[0.025** | **0.975]** | **Dep. Var.** |
| --- | --- | --- | --- | --- | --- | --- | --- |
| **Intercept** | 3.506 | 1.371 | 2.557 | 0.011 | 0.819 | 6.193 | MMSE |
| **Sex[T.M]** | 0.117 | 0.354 | 0.330 | 0.742 | -0.576 | 0.809 | MMSE |
| **fEI 2-4 Hz** | -0.092 | 0.114 | -0.806 | 0.420 | -0.315 | 0.131 | MMSE |
| **fEI 5-7 Hz** | -0.049 | 0.142 | -0.349 | 0.727 | -0.327 | 0.228 | MMSE |
| **fEI 8-13 Hz** | -0.012 | 0.139 | -0.084 | 0.933 | -0.284 | 0.261 | MMSE |
| **fEI 15-30 Hz** | 0.124 | 0.139 | 0.891 | 0.373 | -0.149 | 0.396 | MMSE |
| **fEI 30-70 Hz** | 0.103 | 0.103 | 0.997 | 0.319 | -0.099 | 0.305 | MMSE |
| **Age** | -0.052 | 0.018 | -2.841 | 0.004 | -0.087 | -0.016 | MMSE |
| **Group Var** | 0.538 | 0.416 |  |  |  |  | MMSE |
| **Intercept** | -1.232 | 1.444 | -0.854 | 0.393 | -4.062 | 1.597 | MDS-UPDRS-III |
| **Sex[T.M]** | 0.138 | 0.370 | 0.374 | 0.709 | -0.587 | 0.863 | MDS-UPDRS-III |
| **fEI 2-4 Hz** | -0.054 | 0.130 | -0.412 | 0.681 | -0.309 | 0.202 | MDS-UPDRS-III |
| **fEI 5-7 Hz** | -0.109 | 0.156 | -0.694 | 0.488 | -0.415 | 0.198 | MDS-UPDRS-III |
| **fEI 8-13 Hz** | 0.123 | 0.158 | 0.779 | 0.436 | -0.187 | 0.434 | MDS-UPDRS-III |
| **fEI 15-30 Hz** | 0.011 | 0.159 | 0.069 | 0.945 | -0.300 | 0.322 | MDS-UPDRS-III |
| **fEI 30-70 Hz** | -0.071 | 0.120 | -0.590 | 0.555 | -0.307 | 0.165 | MDS-UPDRS-III |
| **Age** | 0.016 | 0.019 | 0.819 | 0.413 | -0.022 | 0.053 | MDS-UPDRS-III |
| **Group Var** | 0.273 | 0.243 |  |  |  |  | MDS-UPDRS-III |
