# Supplementary material for: Unveiling Cortical Criticality Changes along the Prodromal to the Overt Continuum of Alpha-Synucleinopathy
Source: J Neurosci. 2025 Jul 3;45(31):e1871242025. doi: 10.1523/JNEUROSCI.1871-24.2025 (PMC12311758; doi:10.1523/JNEUROSCI.1871-24.2025)
Supplement: Figure 5-2 — Summary of the Linear Mixed Model (LMM) using as dependent variable clinical scores (i.e., MMSE or MDS-UPDRS-III), as fixed effect the BiS in canonical frequency bands, age, and sex, and as random effect subjects. Download Figure 5-2, DOCX file. [file jneuro-45-e1871242025-s010.docx]

**Figure 5-2:** Summary of the Linear Mixed Model (LMM) using as dependent variable clinical scores (i.e., MMSE or MDS-UPDRS-III), as fixed effect the BiS in canonical frequency bands, age, and sex, and as random effect the subjects.

|  | **Coef.** | **Std.Err.** | **z** | **P>\|z\|** | **[0.025** | **0.975]** | **Dep. Var.** |
| --- | --- | --- | --- | --- | --- | --- | --- |
| **Intercept** | 2.672 | 1.251 | 2.136 | 0.033 | 0.221 | 5.124 | MMSE |
| **Sex[T.M]** | 0.202 | 0.331 | 0.611 | 0.541 | -0.447 | 0.852 | MMSE |
| **BiS 2-4 Hz** | 0.002 | 0.143 | 0.014 | 0.989 | -0.279 | 0.282 | MMSE |
| **BiS 5-7 Hz** | 0.276 | 0.158 | 1.750 | 0.080 | -0.033 | 0.585 | MMSE |
| **BiS 8-13 Hz** | 0.031 | 0.161 | 0.189 | 0.850 | -0.286 | 0.347 | MMSE |
| **BiS 15-30 Hz** | -0.041 | 0.174 | -0.235 | 0.814 | -0.382 | 0.300 | MMSE |
| **BiS 30-70 Hz** | -0.099 | 0.135 | -0.730 | 0.465 | -0.364 | 0.166 | MMSE |
| **Age** | -0.041 | 0.017 | -2.453 | 0.014 | -0.073 | -0.008 | MMSE |
| **Group Var** | 0.469 | 0.368 |  |  |  |  | MMSE |
| **Intercept** | 0.323 | 1.288 | 0.251 | 0.802 | -2.201 | 2.847 | MDS-UPDRS-III |
| **Sex[T.M]** | -0.235 | 0.354 | -0.663 | 0.507 | -0.930 | 0.459 | MDS-UPDRS-III |
| **BiS 2-4 Hz** | -0.123 | 0.158 | -0.776 | 0.438 | -0.432 | 0.187 | MDS-UPDRS-III |
| **BiS 5-7 Hz** | -0.128 | 0.174 | -0.735 | 0.462 | -0.469 | 0.213 | MDS-UPDRS-III |
| **BiS 8-13 Hz** | 0.105 | 0.183 | 0.572 | 0.568 | -0.254 | 0.463 | MDS-UPDRS-III |
| **BiS 15-30 Hz** | -0.169 | 0.195 | -0.867 | 0.386 | -0.552 | 0.214 | MDS-UPDRS-III |
| **BiS 30-70 Hz** | 0.094 | 0.155 | 0.610 | 0.542 | -0.209 | 0.398 | MDS-UPDRS-III |
| **Age** | -0.002 | 0.017 | -0.109 | 0.914 | -0.036 | 0.032 | MDS-UPDRS-III |
| **Group Var** | 0.237 | 0.226 |  |  |  |  | MDS-UPDRS-III |
