# Supplementary material for: Unveiling Cortical Criticality Changes along the Prodromal to the Overt Continuum of Alpha-Synucleinopathy
Source: J Neurosci. 2025 Jul 3;45(31):e1871242025. doi: 10.1523/JNEUROSCI.1871-24.2025 (PMC12311758; doi:10.1523/JNEUROSCI.1871-24.2025)
Supplement: Figure 6-2 — Summary of the Linear Mixed Model (LMM) using as dependent variable clinical scores (i.e., MMSE or MDS-UPDRS-III), as fixed effect the DFA in canonical frequency bands, age, and sex, and as random effect subjects. Download Figure 6-2, DOCX file. [file jneuro-45-e1871242025-s012.docx]

**Figure 6-2**: Summary of the Linear Mixed Model (LMM) using as dependent variable clinical scores (i.e., MMSE or MDS-UPDRS-III), as fixed effect the DFA in canonical frequency bands, age, and sex, and as random effect the subjects.

|  | **Coef.** | **Std.Err.** | **z** | **P>\|z\|** | **[0.025** | **0.975]** | **Dep. Var.** |
| --- | --- | --- | --- | --- | --- | --- | --- |
| **Intercept** | 3.366 | 1.245 | 2.703 | 0.007 | 0.926 | 5.807 | MMSE |
| **Sex[T.M]** | 0.097 | 0.346 | 0.279 | 0.780 | -0.582 | 0.776 | MMSE |
| **DFA 2-4 Hz** | 0.045 | 0.149 | 0.299 | 0.765 | -0.248 | 0.337 | MMSE |
| **DFA 5-7 Hz** | -0.120 | 0.183 | -0.656 | 0.512 | -0.478 | 0.238 | MMSE |
| **DFA 8-13 Hz** | 0.217 | 0.222 | 0.980 | 0.327 | -0.218 | 0.652 | MMSE |
| **DFA 15-30 Hz** | -0.080 | 0.176 | -0.453 | 0.651 | -0.424 | 0.265 | MMSE |
| **DFA 30-70 Hz** | -0.077 | 0.108 | -0.711 | 0.477 | -0.287 | 0.134 | MMSE |
| **Age** | -0.049 | 0.017 | -2.965 | 0.003 | -0.082 | -0.017 | MMSE |
| **Group Var** | 0.503 | 0.391 |  |  |  |  | MMSE |
| **Intercept** | -0.256 | 1.241 | -0.207 | 0.836 | -2.688 | 2.175 | MDS_UPDRS-III |
| **Sex[T.M]** | 0.075 | 0.361 | 0.206 | 0.836 | -0.634 | 0.783 | MDS_UPDRS-III |
| **DFA 2-4 Hz** | 0.038 | 0.165 | 0.232 | 0.816 | -0.286 | 0.363 | MDS_UPDRS-III |
| **DFA 5-7 Hz** | 0.027 | 0.212 | 0.126 | 0.900 | -0.389 | 0.442 | MDS_UPDRS-III |
| **DFA 8-13 Hz** | 0.099 | 0.239 | 0.412 | 0.680 | -0.370 | 0.568 | MDS_UPDRS-III |
| **DFA 15-30 Hz** | -0.250 | 0.194 | -1.289 | 0.198 | -0.631 | 0.130 | MDS_UPDRS-III |
| **DFA 30-70 Hz** | 0.004 | 0.123 | 0.031 | 0.975 | -0.238 | 0.246 | MDS_UPDRS-III |
| **Age** | 0.003 | 0.017 | 0.156 | 0.876 | -0.030 | 0.036 | MDS_UPDRS-III |
| **Group Var** | 0.199 | 0.208 |  |  |  |  | MDS_UPDRS-III |
