# Supplementary material for: Unveiling Cortical Criticality Changes along the Prodromal to the Overt Continuum of Alpha-Synucleinopathy
Source: J Neurosci. 2025 Jul 3;45(31):e1871242025. doi: 10.1523/JNEUROSCI.1871-24.2025 (PMC12311758; doi:10.1523/JNEUROSCI.1871-24.2025)
Supplement: Figure 7-1 — Summary of the five Linear Mixed Model (LMM) using as dependent variable the fEi in a frequency band (i.e., delta, theta, alpha, beta, and gamma), as fixed effect the nodal wPLI measures (i.e., strength, clustering coefficient, and eigen vector centrality) in each canonical frequency bands, age, and sex, and as random effect subjects. Download Figure 7-1, DOCX file. [file jneuro-45-e1871242025-s013.docx]

**Figure 7-1:** Summary of the five Linear Mixed Model (LMM) using as dependent variable the fEi in a frequency band (i.e., delta, theta, alpha, beta, and gamma), as fixed effect the nodal wPLI measures (i.e., strength, clustering coefficient, and eigen vector centrality) in each canonical frequency bands, age, and sex, and as random effect the subjects.

|  | **Coef.** | **Std.Err.** | **z** | **P>\|z\|** | **[0.025** | **0.975]** | **Dep. Var.** |
| --- | --- | --- | --- | --- | --- | --- | --- |
| **Intercept** | -3.673 | 1.181 | -3.111 | 0.002 | -5.986 | -1.359 | fEI 2-4 Hz |
| **Sex[T.M]** | 0.580 | 0.311 | 1.865 | 0.062 | -0.029 | 1.189 | fEI 2-4 Hz |
| **Eigen Vector Centrality** | 0.059 | 0.116 | 0.506 | 0.613 | -0.169 | 0.286 | fEI 2-4 Hz |
| **Strength** | 0.288 | 1.435 | 0.201 | 0.841 | -2.524 | 3.101 | fEI 2-4 Hz |
| **Clustering Coefficient** | -0.212 | 1.423 | -0.149 | 0.882 | -3.000 | 2.577 | fEI 2-4 Hz |
| **Age** | 0.045 | 0.016 | 2.851 | 0.004 | 0.014 | 0.076 | fEI 2-4 Hz |
| **Group Var** | 0.248 | 0.260 |  |  |  |  | fEI 2-4 Hz |
| **Intercept** | -3.799 | 1.112 | -3.417 | 0.001 | -5.979 | -1.620 | fEI 5-7 Hz |
| **Sex[T.M]** | 0.430 | 0.291 | 1.480 | 0.139 | -0.140 | 1.000 | fEI 5-7 Hz |
| **Eigen Vector Centrality** | 0.069 | 0.098 | 0.707 | 0.479 | -0.123 | 0.261 | fEI 5-7 Hz |
| **Strength** | 1.860 | 1.619 | 1.149 | 0.251 | -1.314 | 5.034 | fEI 5-7 Hz |
| **Clustering Coefficient** | -1.457 | 1.611 | -0.905 | 0.366 | -4.614 | 1.699 | fEI 5-7 Hz |
| **Age** | 0.049 | 0.015 | 3.245 | 0.001 | 0.019 | 0.078 | fEI 5-7 Hz |
| **Group Var** | 0.421 | 0.383 |  |  |  |  | fEI 5-7 Hz |
| **Intercept** | -0.132 | 1.171 | -0.113 | 0.910 | -2.428 | 2.164 | fEI 8-13 Hz |
| **Sex[T.M]** | -0.667 | 0.312 | -2.141 | 0.032 | -1.278 | -0.056 | fEI 8-13 Hz |
| **Eigen Vector Centrality** | -0.147 | 0.109 | -1.344 | 0.179 | -0.361 | 0.067 | fEI 8-13 Hz |
| **Strength** | 0.988 | 1.902 | 0.520 | 0.603 | -2.740 | 4.716 | fEI 8-13 Hz |
| **Clustering Coefficient** | -0.633 | 1.908 | -0.332 | 0.740 | -4.373 | 3.107 | fEI 8-13 Hz |
| **Age** | 0.010 | 0.016 | 0.645 | 0.519 | -0.020 | 0.041 | fEI 8-13 Hz |
| **Group Var** | 0.463 | 0.378 |  |  |  |  | fEI 8-13 Hz |
| **Intercept** | -1.620 | 1.267 | -1.279 | 0.201 | -4.103 | 0.862 | fEI 15-30 Hz |
| **Sex[T.M]** | -0.408 | 0.331 | -1.233 | 0.218 | -1.056 | 0.241 | fEI 15-30 Hz |
| **Eigen Vector Centrality** | 0.096 | 0.106 | 0.904 | 0.366 | -0.112 | 0.305 | fEI 15-30 Hz |
| **Strength** | 1.618 | 0.788 | 2.052 | 0.040 | 0.073 | 3.163 | fEI 15-30 Hz |
| **Clustering Coefficient** | -1.705 | 0.792 | -2.151 | 0.031 | -3.258 | -0.152 | fEI 15-30 Hz |
| **Age** | 0.028 | 0.017 | 1.637 | 0.102 | -0.005 | 0.061 | fEI 15-30 Hz |
| **Group Var** | 0.606 | 0.455 |  |  |  |  | fEI 15-30 Hz |
| **Intercept** | -2.539 | 0.875 | -2.903 | 0.004 | -4.254 | -0.825 | fEI 30-70 Hz |
| **Sex[T.M]** | 0.519 | 0.237 | 2.190 | 0.028 | 0.055 | 0.984 | fEI 30-70 Hz |
| **Eigen Vector Centrality** | -0.092 | 0.098 | -0.936 | 0.349 | -0.285 | 0.101 | fEI 30-70 Hz |
| **Strength** | 2.057 | 0.542 | 3.797 | 0.000147 | 0.995 | 3.119 | fEI 30-70 Hz |
| **Clustering Coefficient** | -2.527 | 0.534 | -4.731 | 0.000002 | -3.574 | -1.480 | fEI 30-70 Hz |
| **Age** | 0.030 | 0.012 | 2.516 | 0.012 | 0.007 | 0.053 | fEI 30-70 Hz |
| **Group Var** | 0.066 | 0.182 |  |  |  |  | fEI 30-70 Hz |
